# Correction: Ecological responses to blue water MPAs

**DOI:** 10.1371/journal.pone.0238558

**Published:** 2020-08-27

**Authors:** The PLOS ONE Staff

## Notice of republication

This article was republished on August 12, 2020 to replace a corrupted supporting information file. No changes were made to the rest of the article. The publisher apologizes for the error. The originally published, uncorrected article and the republished, corrected article are provided here for reference.

## Supporting information

S1 File Originally published, uncorrected article. (PDF)

S2 File Republished, corrected article. (PDF)
