# Supplementary material for: Ecological responses to blue water MPAs
Source: PLoS One. 2020 Jul 8;15(7):e0235129. doi: 10.1371/journal.pone.0235129 (PMC7343131; doi:10.1371/journal.pone.0235129)
Supplement: S1 File — (PDF) [file pone.0235129.s001.pdf]

Supporting Information

Ecological responses to blue water MPAs

E. Gilman, M. Chaloupka, M. Fitchett, D. Cantrell, M. Merrifield

S1. Data Sources

S1.1. Fisheries Observer Data

Data were obtained from the U.S. National Marine Fisheries Service observer program for Hawaii's pelagic longline fishery making deep sets, with ≥ 15 hooks between two floats (NMFS, 2004). There were 4,942 trips in the study sample, during which 146,330,902 hooks were deployed during 64,611 sets. These trips were made by 202 vessels. We excluded records that had not yet been validated and approved at the time of accessing the observer program dataset and for which earlier trips had not yet been processed. This latter protocol was implemented because observed trips with protected species interactions are prioritized for processing (pers. comm., Eric Forney, 28 March 2016, NMFS Pacific Islands Regional Office), where trips that were processed in advance of earlier trips are biased.

From 1994 through August 2003, observers estimated lengths to the nearest whole foot, after which they measured lengths to the nearest cm. From August 2003 through February 2006, observers recorded lengths of all tunas, billfishes and sharks. Since February 2006 observers have recorded the length of every third caught fish regardless of species, excluding rays (NMFS, 2017c). Fork lengths for sharks collected by observers either by making visual estimates to the nearest whole foot or by measuring to the nearest cm, and curved carapace length for sea turtles measured to the nearest cm were used in the assessment for the full study period. Eye-to-fork length data for billfishes and fork length data for other teleosts and pelagic stingrays, measured by observers to the nearest cm, were used from August 2003 to the end of the study period. These protocols were selected to maximize sample sizes of length data using the same length measurement method.

S1.2. Environmental Time Series Data

We focused on using macro-scale ocean/climate indicators as potential environmental drivers known to affect pelagic fish productivity (Newman et al., 2016, Free et al., 2019). We sourced spatially averaged, monthly sea surface temperature (SST) data for the Palmyra/Kingman and Johnston regions using the ERSSTv5 index (Huang et al., 2017). Then, a GAMM with an AR(1) within month error structure was fitted to the monthly averaged data using the `mgcv` R package (Wood, 2006) to derive the expected annual SST time series. Fig. S1 presents expected annual SST time series for the Palmyra Atoll and Johnston Atoll regions.

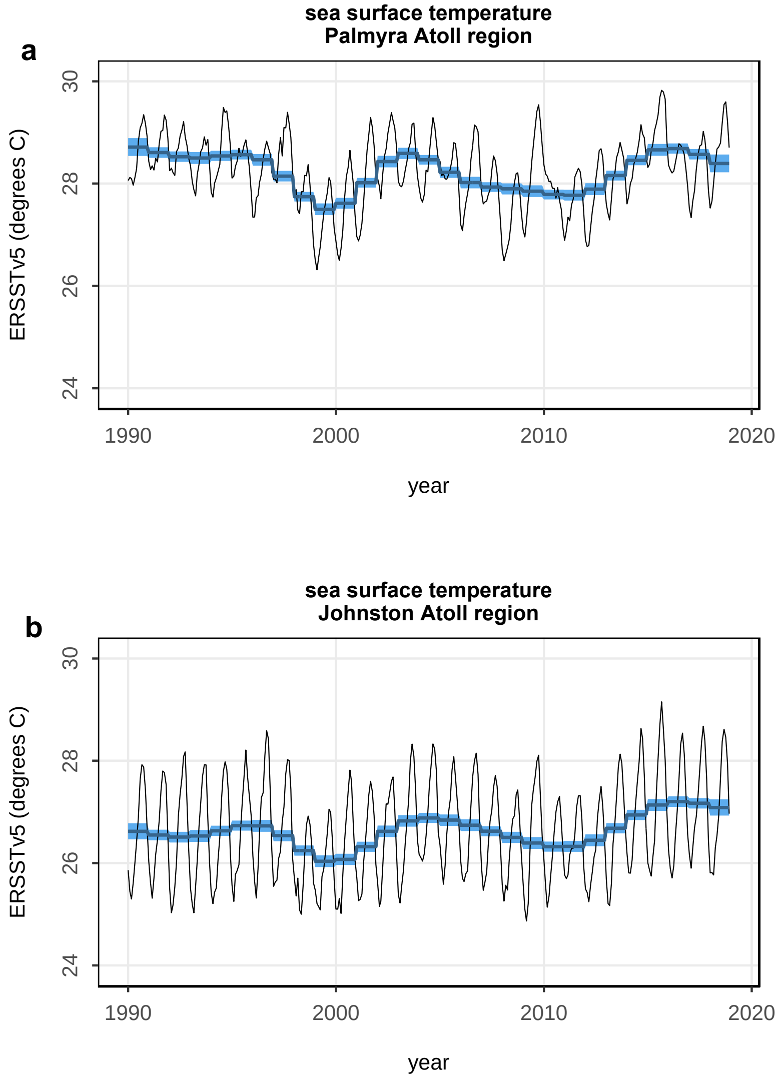

Fig. S1. ERSSTv5 (Huang et al., 2017) - sourced monthly SST data since 1990 for the (a) Palmyra Atoll region, and (b) Johnston Atoll region with a GAMM smooth curve (95% confidence band) superimposed to highlight the underlying long-term trend to derive the annualized SST estimates. The GAMM included an AR(1) within month correlated error structure.

We sourced the monthly Pacific Decadal Oscillation (PDO) index using the `rpdo` R Package (Thorley et al., 2018). The PDO is a regional climate index based on cyclical variations in North Pacific sea surface temperature (Newman et al., 2016). The monthly PDO index was annualized using the method used to annualize the ERSST data.

We sourced the revised bimonthly Multivariate ENSO Index (MEI) data (Zhang et al., 2019; NOAA, 2020), which we annualized using the method used to annualize the ERSST data. Annualized time series were necessary in order to match the same temporal resolution as the annual trends in expected catch rates used to derive the annual counterfactual predictions.

S1.3. Economic Time Series Data

We sourced the annual FAO Fish Price Index (Tveteras et al., 2012) time series data for (1) all capture fisheries from 1990-2018 and (2) for tuna species only for the same time period (see

Fig. S2). These quantity-based indices of global seafood prices reflect fish scarcity and perhaps regional demand and supply shocks that might influence fisher behavior (Tveteras et al., 2012) — and so might have a bearing on our counterfactual predictions for regional tuna catch rates.

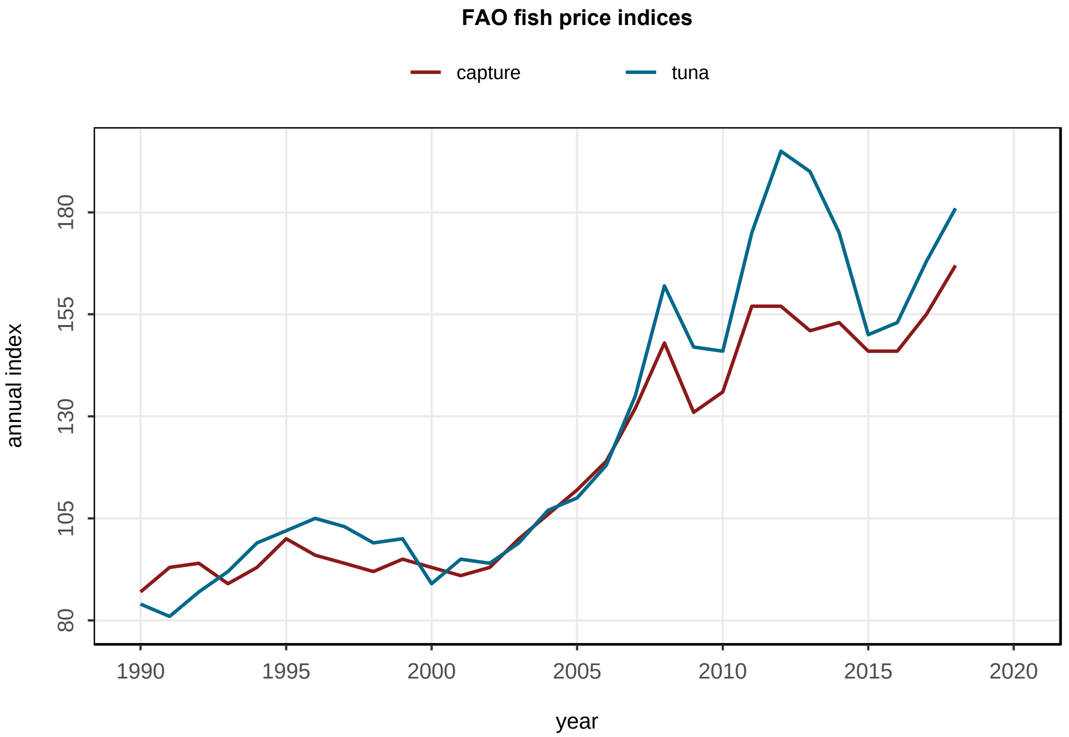

Fig. S2. Annual FAO Fish Price indices for “all capture fisheries” and the “tuna” species only. Data provided courtesy of the FAO (Rome: Adrienne Egger) and the Norwegian Seafood Council.

S1.4. Bathymetric Depth Data

We sourced the bathymetric depth (depth to seafloor: *m*) for the geolocation of each set using the `marmap` R package (Pante and Simon-Bouhet, 2013).

S1.5. Trophic Level Data

Trophic level values were obtained from the central north Pacific Ocean Ecopath model of Choy et al. (2016), accounting for ontogenetic stage (juvenile vs. adult) where possible, or otherwise from FishBase (Froese and Pauly, 2019). To determine ontogenetic stage, estimates of length at first maturity (L_m) were obtained from FishBase (Froese and Pauly, 2019), Okamoto and Bayliff (2003), and Molony (2005).

S2. Data Standardization Models

The following predictors were considered for inclusion as terms in data standardization models:

- Unique vessel;
- Vessel overall length;
- Soak duration (elapsed time between setting the first hook and retrieving the last hook);
- Geospatial location at the start of the set;

- Distance from port. We calculated the great circle distance (*km*) of each set from the port of Honolulu (the port of origin for this fishery) using the haversine function in the `geosphere` R package (Hijmans, 2019);
- Season (quarter) at the start of the set, modelled using cyclic cubic regression splines to account for the cyclical behaviour of those terms (Wood, 2006);
- Time-of-day at the start of the set, modelled using cyclic cubic regression splines (Wood, 2006);
- Number of hooks per set;
- Number of hooks between two floats as a 3-level factor of 15-24, 25-29, and ≥30 hooks between floats;
- Hook shape (circle, tuna, J, mix);
- Bait species (saury, sardine, other, mix);
- Leader material (wire, monofilament, other);
- Blue-dyed vs. untreated bait;
- PDO;
- Seafloor depth at the location of the start of the set;
- Zone (three control zones, three treatment zones); and
- Other coexisting regional area closures, described below.

The inclusion of coexisting regional management policy changes that affect the entire fishery and not just those fishing around a no-take MPA or exclusion zone has been shown to be important in evaluating the impact of marine reserves (Smith et al., 2006). So, we included dummy variable indicators for the following 5 fishery-wide management policy change events of area closures for Hawaii tuna longline vessels. (1) FKW: During the study period, a closure occurred from 24 July – 31 Dec. 2018 in an area south of the Main Hawaiian Islands due to interactions with false killer whales (NMFS, 2018). (2) NWHI1: A U.S. Presidential Proclamation, signed 15 June 2006, established the Northwestern Hawaiian Islands Marine National Monument (later renamed Papahānaumokuākea Marine National Monument), which prohibits pelagic longline fishing within 50 nm of the Northwestern Hawaiian Islands. (3) NWHI2: A subsequent Presidential Proclamation, signed 26 August 2016, extended the monument's seaward margin west of 163° West longitude out to 200 nm (Executive Office of the President, 2006, 2016). (4) WCPFC: Closures occurred for Hawaii's tuna longline fishery during parts of 2015-2017 in the Western and Central Pacific Fisheries Commission (WCPFC) Convention Area and (5) IATTC closures occurred during parts of 2013-2017 in the Inter-American Tropical Tuna Commission (IATTC) Convention Area (the latter closure was limited to Hawaii tuna longline vessels over 24 m in overall length) when bigeye tuna catch limits were reached (NMFS, 2013b, 2014, 2015b,c, 2016a, 2017a,b).

Several additional variables that significantly explain catch rates and length frequency distribution were considered but excluded due either to low variability in the data sample (e.g., lightstick use) or data quality constraints (e.g., hook minimum width, captain name).

Catch rates*:* The species-specific catch rate models were varying coefficient geoGAMMs (Kammann and Wand, 2003) with negative binomial likelihood and georeferenced set locations modelled as a 2D Gaussian process with Matérn covariance kernel (Gelfand and Schliep, 2016). The random effect structure (intercepts-only) in each model was the unique identity of the 202 individual vessels to account for any correlated or vessel-specific heterogeneity in catch rates not accounted for by the covariates. The geoGAMMs were varying coefficient models because the temporal trends (annual effect) were modelled separately within each of the treatment and reference zones but within the same spatio-temporal model (Hastie and Tibshirani, 1993).

Cubic smoothing splines (Wood, 2016) were used to account for possible nonlinear functional form of the covariates such as fishing effort (log of hooks set), year, seafloor depth, distance to port of origin for each vessel, overall length of vessel, soak time and regional ocean/climate metrics such as the monthly PDO index. Seasonal (month) and time-of-day effects were modelled using cyclic cubic regression splines to account for the cyclical behaviour of those terms (Wood, 2006). Factor terms included bait type, hook type, number of hooks per float, whether blue-dyed bait was used, and the five dummy variable indicators to mark regional management policy changes.

All geoGAMMS were fit using the `mgcv` R package (Wood, 2006) with model fit assessed using the posterior simulation based diagnostic tools in the `mgcViz` R package (Fasiolo et al., 2019). We then used `mgcViz` and the `ggplot2` R package (Wickham, 2016) for visualization of all geoGAMM results with the *viridis* color palette from the `colorspace` R package (Zeileis et al., 2019) used for all spatial effects plots. The `cowplot` R package (Wilke, 2019) was used for all multi-panel plot layouts. We then extracted the predicted conditional effects from these models suitable for visualization and post-processing using the `ggeffects` R package (Lüdecke, 2018) — these predicted effects for each zone for each year for each species were used to derive the counterfactual predictions for the standardized catch rate metric.

Species were selected for the assessment of the standardized catch rate (relative abundance) response based on whether they: (1) made up ≥10% of the catch in sets made in treatment areas, and/or in sets made in control zones, or (2) are a species of conservation concern with a sufficient sample size. For the former, this included albacore (*Thunnus alalonga*), bigeye (*T. obesus*) and yellowfin tunas (*T. albacares*); blue shark (*Prionace glauca*); and longnose lancetfish (*Alepisaurus ferox*). For the latter, this included striped marlin (*Kajikia audax*) as well as already included blue shark.

***Mean length**:* Mean length assessments were conducted for bigeye and yellowfin tunas. The species-specific expected length model was a GLMM with Gaussian likelihood with each of the 4942 fishing trips as the random effect. The model comprised all conditional effects for "zone (n=6: 3 reference, 3 treatment) * species (n=2) * intervention (pre-2009 or otherwise)". We explored other model likelihoods such as *lognormal* using the `gamlss` R package (Rigby et al., 2005) but a Gaussian model was the best comparative fit based on the generalized Akaike Information Criterion (Rigby et al., 2005). This was a large memory-resident data set (>600k observations) and so for computational reasons and compatibility with `ggeffects` used for extraction of predictions, we fitted this Gaussian GLMM using the TMB computation engine (Kristensen et al., 2016) via the `glmmTMB` R interface (Brooks et al., 2017). The model-predicted conditional effects were used to derive the counterfactual predictions for this standardized metric.

***Mean TLc**:* Set-specific mean trophic level of the catch (TLc) was calculated using a landings-based indicator (Shannon et al., 2014) based on the catch of each species in a set and the species-specific and where possible the ontogenetic stage-specific trophic level. The set-specific mean trophic level data were then modelled using a varying coefficient GAMM with Gaussian likelihood with the 202 individual vessels as the intercepts-only random effect. The temporal (annual trend) was zone-specific where the 2 zones here were now: (1) the combined reference or control zones, and (2) the combined treatment zones. Other than the parametric zone term there was no explicit spatial effect considered for this response metric. Similar to the catch rate models, the predictors included fishing effort (log of hooks set), year, season (quarter), time-of-day, soak time, length of vessel, bait type, hook type, hooks per float, whether blue-dyed bait was used and indicators to mark 5 additional fishery-wide management policy changes. Similar to catch models, this model was fitted using the `mgcv` R package (Wood,

2006) with model fit assessed using simulation based predictive checks (Fasiolo et al., 2019). The model-predicted conditional effects were then used to derive the counterfactual predictions for this standardized metric. The time series for mean annual TLc in KP 110 was 1997-2015, and for J110 and J260 combined was 1995-2018. Sets with 0 catch were eliminated from the study sample for this component.

Species diversity metrics*:* The overall catch species pool comprised 97 distinct taxa of greatly varying abundance so we explored 3 well-known metrics of taxonomic diversity for the annual catch using the `iNEXT` R package: (1) species richness, (2) Shannon-Wiener Index of diversity *H'* (also referred to as the Shannon Index), and (3) Simpson diversity (Hsieh et al., 2016). These standardized diversity metrics were then used to compare annual catch species diversity between the 2 treatment zones and a combined reference zone to explore any zone-specific effort and sample coverage issues using rarefaction and extrapolation approaches (Hsieh et al 2016).

Species richness is extremely sensitive to sample size and species abundance distribution (evenness). The less even the relative abundance of species in a community is, the larger the proportion of relatively rarer species within that system will be detected with more sampling effort (e.g., Heck et al., 1975; Lawton et al., 1998). Other species-level biodiversity indices, such as the Shannon index and inverse Simpson index, are similarly affected by the relative abundance of common species as well as by species richness (Hill, 1973; Brewer and Williamson, 1994). H' increases as both the species richness and the evenness of a community increase (Shannon, 1948; Magurran, 2004). The Shannon index is affected by the relative abundance of common species as well as by species richness, which is extremely sensitive to sample size and species abundance distribution (evenness) (Hill, 1973; Heck et al., 1975; Brewer and Williamson, 1994; Lawton et al., 1998).

Effort standardization explicitly accounted for variability in sampled effort by study zone. We explored the importance of effort standardization to account for any zone-specific inadequate sample sizes in estimating the Shannon index species diversity response. We employed rarefaction (interpolation to smaller sample sizes, the expected species diversity response using standardized subsamples) and extrapolation (prediction to a larger sample size) following methods described in Hsie et al. (2016) to explore how important standardizing effort was for the Shannon index response for control and treatment zones. We also explored the effect of sample size on two other species-level biodiversity metrics of species richness and Simpson diversity metric. Fig. S3 shows that, of the three species diversity metrics, sample size had the largest effect on the species richness response (both rarefied to a smaller sample and extrapolated to a larger sample), and there was extremely large uncertainty in the extrapolated species richness response in the Kingman/Palmyra treatment zone. In all three indices, a larger sample size would have had the largest effect on the response in the Palmyra/Kingman treatment zone. Additional sampling effort would have minimally affected the Shannon index response in the control and treatment zones, where a larger sample size would have had the largest effect on the response in the Palmyra/Kingman treatment zone. Thus, this exploration determined that, for the Shannon index response, the geoGAMM effort standardization was most important for the Kingman/Palmyra treatment zone, and for this species diversity metric response, sample sizes were largely adequate as there would have been minimal effect from extrapolation to a larger sample size. Therefore, sample sizes were found to be sufficient for our modelling purposes with standardized diversity similar between the 2 treatment zones and lower than the reference zone for any given sample size (Fig. S3). Based on this review we chose the Shannon index for further zone-specific analysis at the set-specific level accounting for set-specific sampling effort (in this case the number of hooks deployed in each set).

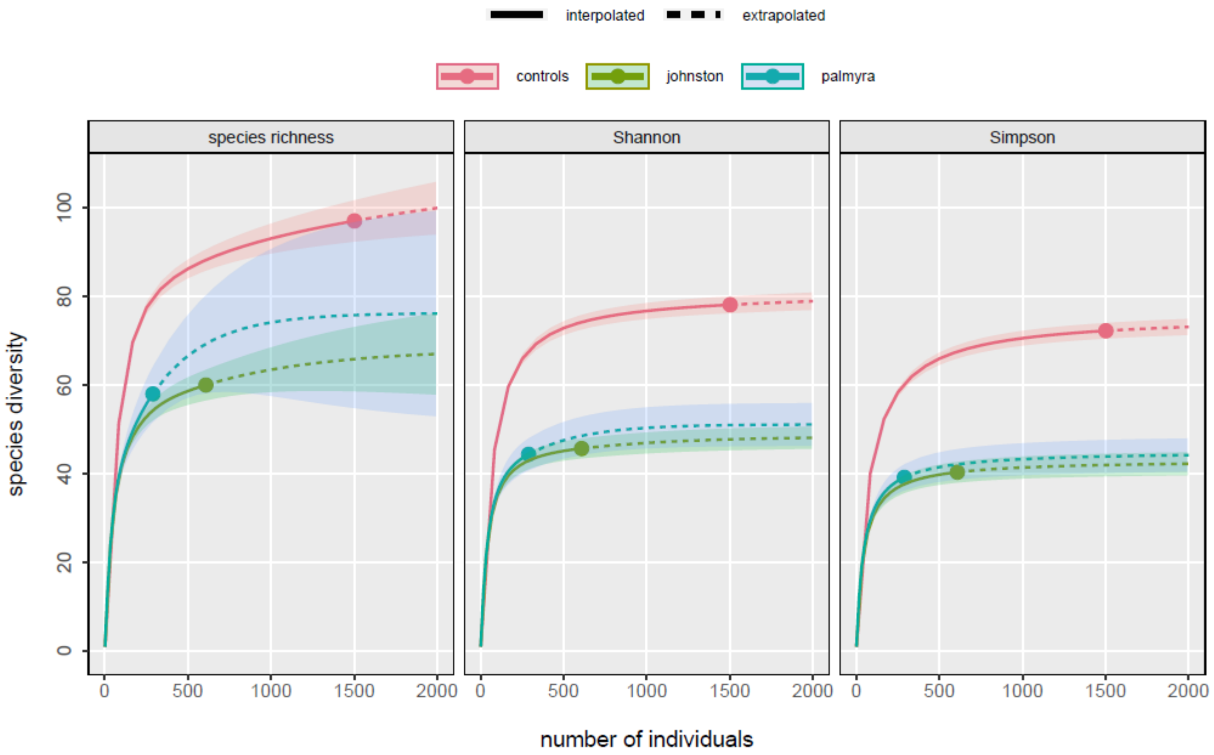

Fig. S3. Sample-size based rarefaction and extrapolation sampling curves with 95% CIs for control and treatment zones for three species-level biodiversity metrics.

We calculated the Shannon index for each of the 64,408 sets with catch > 0 since 1999 using the `vegan` R package (Oksanen et al., 2019) to assess the temporal effect of displaced effort on species diversity — there was insufficient annual catch records for all treatment and reference zones prior to 1999. The set-specific Shannon index data were then modelled using a varying coefficient GAMM with Gaussian likelihood with the 202 individual vessels as the intercepts-only random effect. The temporal (annual trend) was zone-specific for the combined treatment zones compared to each of the 3 references or control zones. Other than the zone term there was no explicit spatial effect considered here. The predictors included fishing effort (log of hooks set), year, season (quarter), time-of-day, soak time, length of vessel, bait type, hook type, hooks per float, whether blue-dyed bait was used and dummy indicators to mark 2 additional fishery-wide management policy changes that occurred post-1999 (IATTC, initial NWHI Marine National Monument designation). The model-predicted conditional effects were used to derive the counterfactual predictions for this standardized metric.

S3. Gaussian geoGAMM – Bigeye Tuna Standardized Catch Rate

Figs. S4-S6 present outcomes of the Gaussian geoGAMM for the bigeye tuna standardized catch rate.

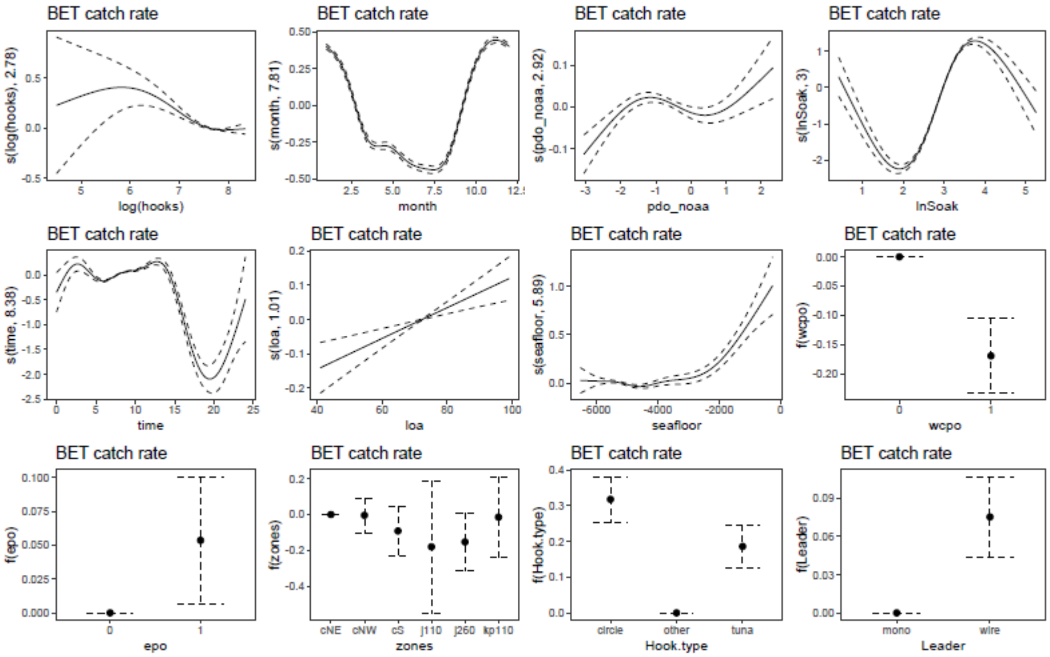

Fig. S4. Significant predictors of the bigeye tuna standardized catch rate geoGAMM. BET=bigeye tuna; Month=month of the start of the set; Hooks=number of hooks per set; PDO= Pacific Decadal Oscillation; Soak=duration that gear remained in the water (elapsed time between the first hook was set and the last hook was retrieved); Time=time-of-day at the start of the set; LOA=vessel length overall – the total length of the vessel's hull; Seafloor=depth of the seafloor at the start of the set; WCPO=periods that the fishery was closed in the western and central Pacific Ocean due to reaching a bigeye tuna catch limit of the Western and Central Pacific Fisheries Commission; EPO=periods that the fishery was closed in the eastern Pacific Ocean due to reaching a bigeye tuna catch limit of the Inter-American Tropical Tuna Commission; Zones=northeast control (cNE), northwest control (cNW), southern control (cS), 110 nm treatment zone around Johnston Atoll (J110), 260 nm treatment zone around Johnston Atoll (J260), 110 nm treatment zone around Kingman Reef and Palmyra Atoll (kp110); Hook type= circle hook, tuna hook, J hook, or mix; Leader=leader material of either monofilament, wire or other.

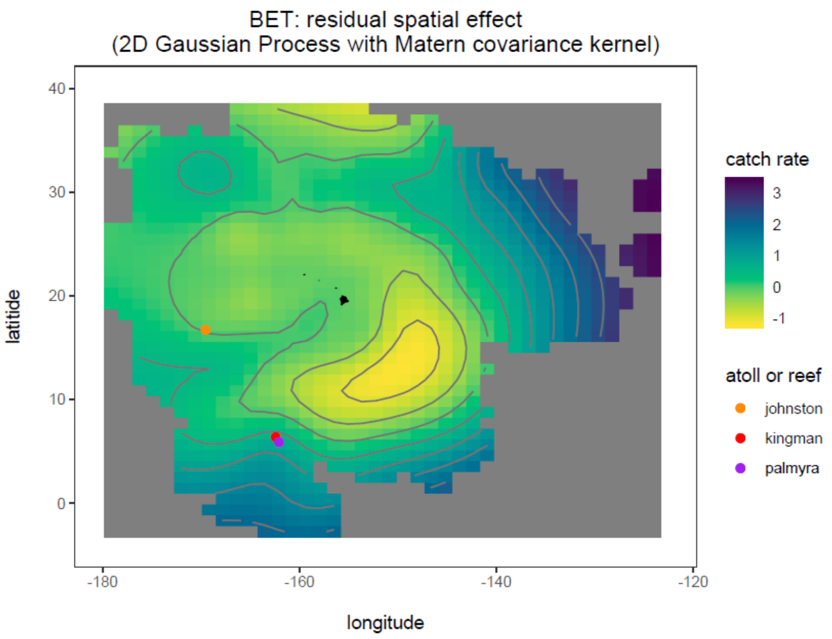

Fig. S5. Residual spatial effect from the bigeye tuna standardized catch rate geoGAMM.

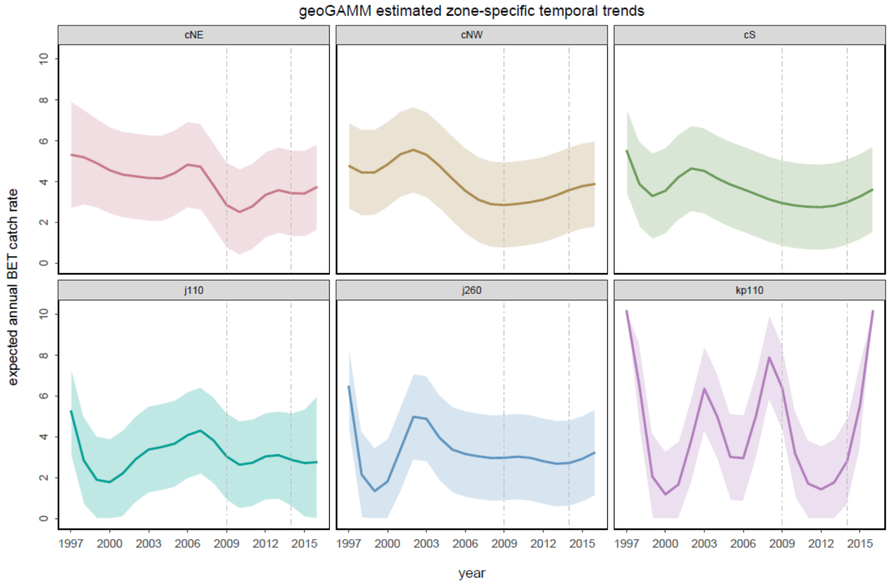

Fig. S6. Temporal trend in standardized bigeye tuna catch rate for six study zones from a Gaussian geoGAMM. Shaded areas are 95% uncertainty intervals. cNE=northeast control zone, cNW=northwest control zone, cS=southern control zone, j110=Johnston Atoll 0-110 nm counterfactual treatment zone, j260=Johnston Atoll 0-260 nm counterfactual treatment zone, kp110= Kingman Reef and Palmyra Atoll 0-110 nm counterfactual treatment zone.

S4. Non-Significant Counterfactual Predictions of Response to MPAs

S4.1. Mean Length Responses

Figs. S7a and S7b present the counterfactual mean length predictions for KP110 and for the combined treatment zones at Johnston (J110 and J260), respectively. In the top panel, the solid

line is the “observed” (predicted from the geoGAMM) mean length response, and the dashed line is the Bayesian state-space structural time series model fit to generate the counterfactual mean length prediction. The middle panel is the pointwise difference between the two curves of the first panel, demonstrating the temporal dynamics of the apparent response to the MPA intervention. The bottom panel shows the cumulative response since the MPA closure. In the top panel, the dashed line to the left of the vertical line is the modeled prediction, and to the right of the vertical line is the counterfactual prediction post-intervention (i.e., what the response would have been in the treatment zone had there not been an intervention). The vertical dashed line indicates the beginning of the counterfactual prediction period. Shaded areas are 95% uncertainty intervals (95% UIs) of the counterfactual prediction.

There were no causal effects on bigeye or yellowfin tuna mean lengths attributable to the 50 nm MPA closure within KP110 or to the two MPA closures within J260. For KP110, the yellowfin mean length impact was minor, gradual and permanent, while the bigeye impact was minor and variable (Fig. S7a). For the Johnston treatment zones, the yellowfin impact was also minor, gradual and permanent, while the bigeye impact was minor and variable (Fig. S7b).

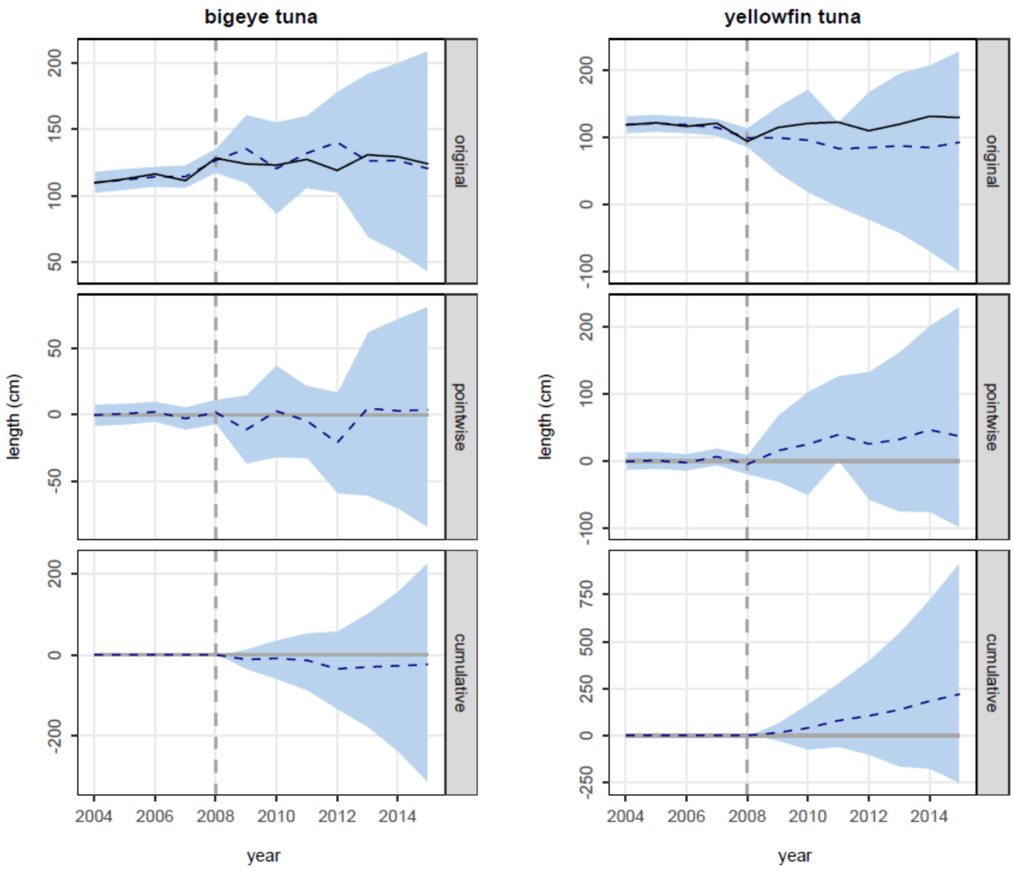

Fig. S7a. Counterfactual prediction for bigeye and yellowfin tuna annual mean length responses to a 50 nm MPA around Kingman Reef and Palmyra Atoll.

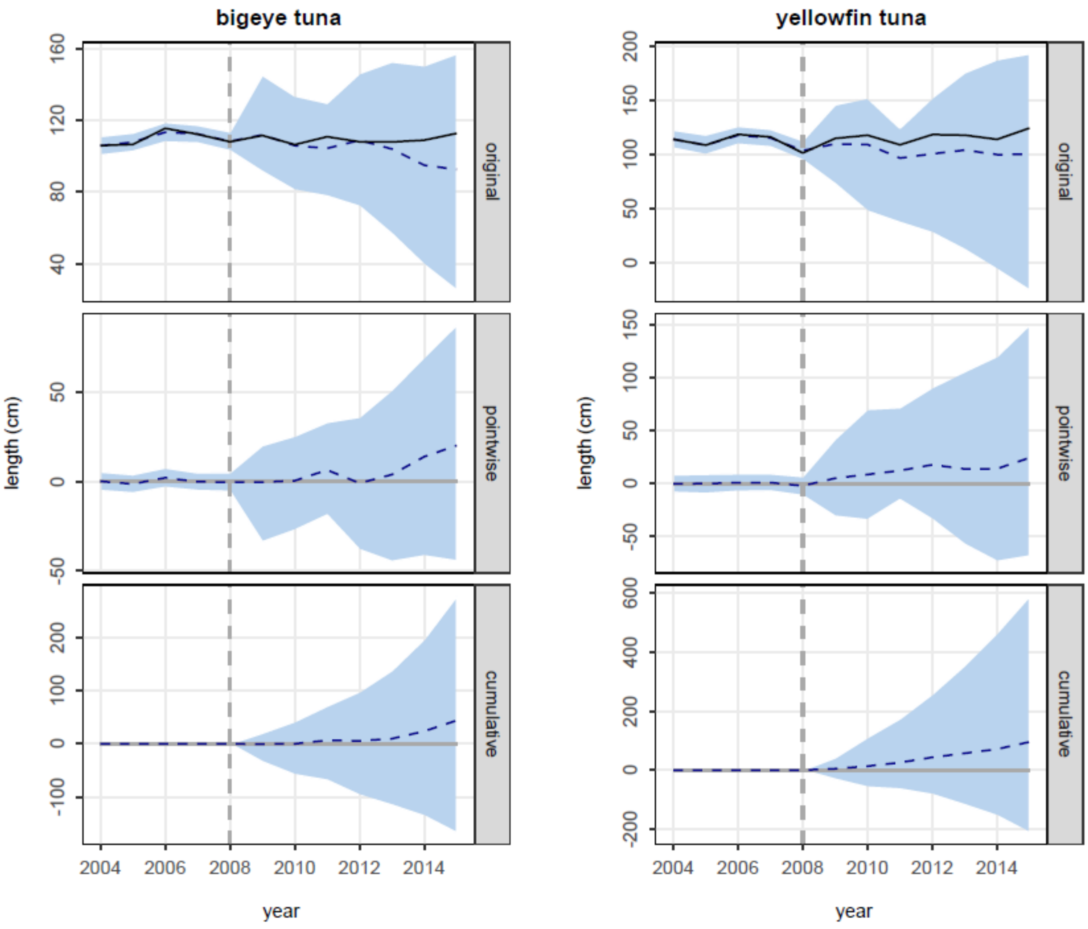

Fig. S7b. Counterfactual prediction for bigeye and yellowfin tuna annual mean length responses to 50 nm and 200 nm MPAs around Johnston Atoll.

S4.2. Mean TLc Response

Fig. S8a presents the counterfactual mean TLc predictions for KP110 and Fig. S8b for combined J110 and J260. There were gradual but temporary mean TLc responses to the MPAs. These effects were not significant - the 95% UIs overlap the zero-baseline post 2009 in pointwise (middle) panels of Fig. S8.

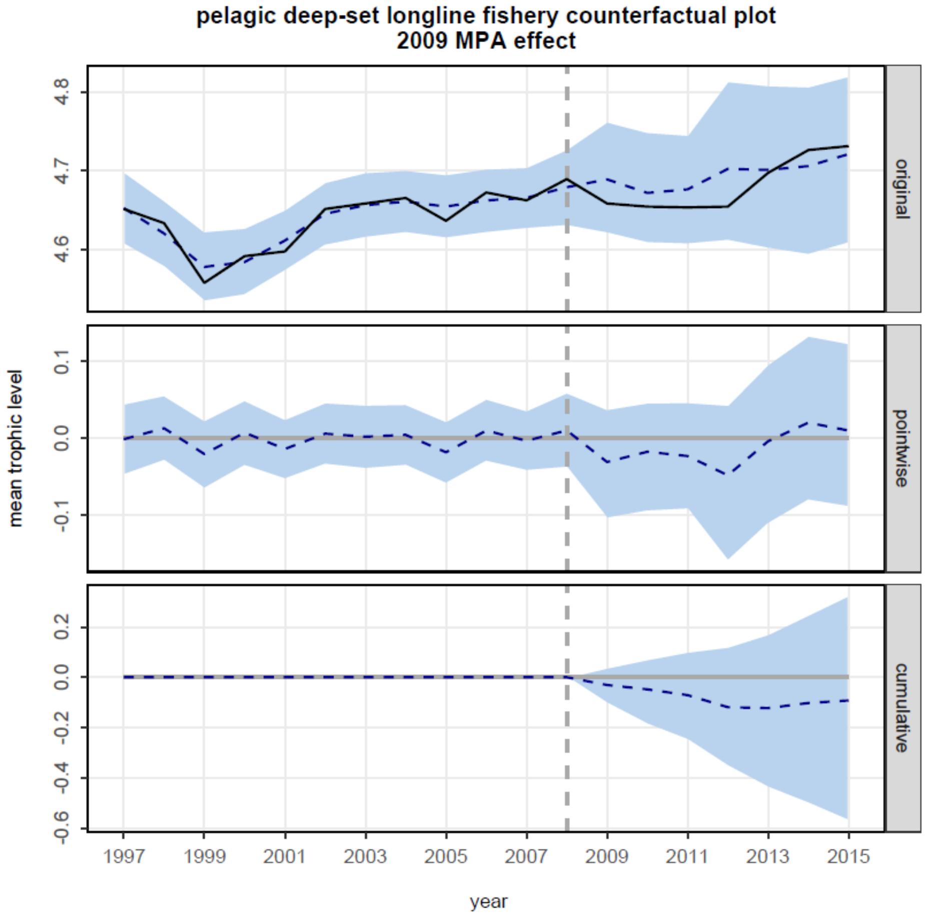

Fig. S8a. Counterfactual prediction for annual mean TLc response to a 50 nm MPA around Kingman Reef and Palmyra Atoll.

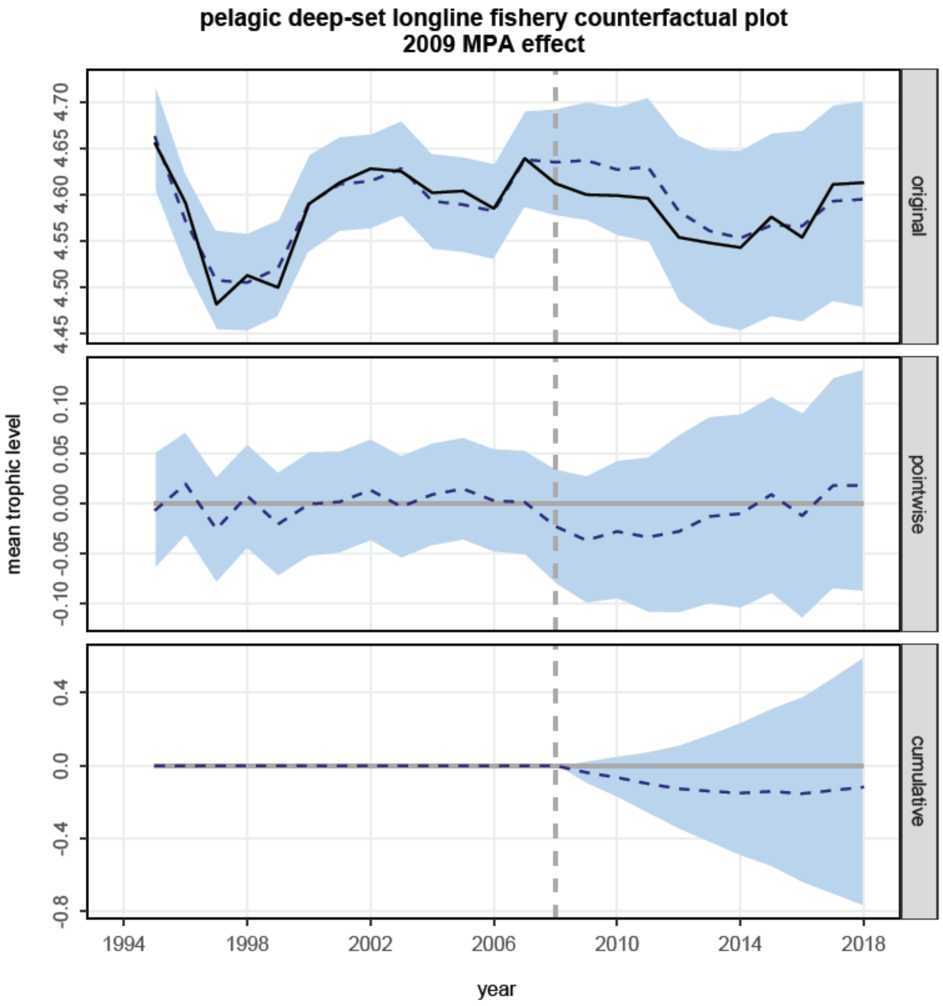

Fig. S8b. Counterfactual prediction for annual mean TLc response to 50 and 200 nm MPAs around Johnston Atoll.

S4.3. Shannon Diversity Index Response

Fig. S9 presents the counterfactual prediction for the standardized mean annual Shannon index within both KP110 and J110. There was a gradual but temporary response to the MPAs from 2009 onwards, with a very weak inference of a very small relative increase in annual mean Shannon H' following establishment of the MPAs, but this effect was not significant - the expected Shannon index in the set-specific catches within the treatment zones around both Kingman/Palmyra and Johnston would have remained the same with or without the establishment of the MPAs.

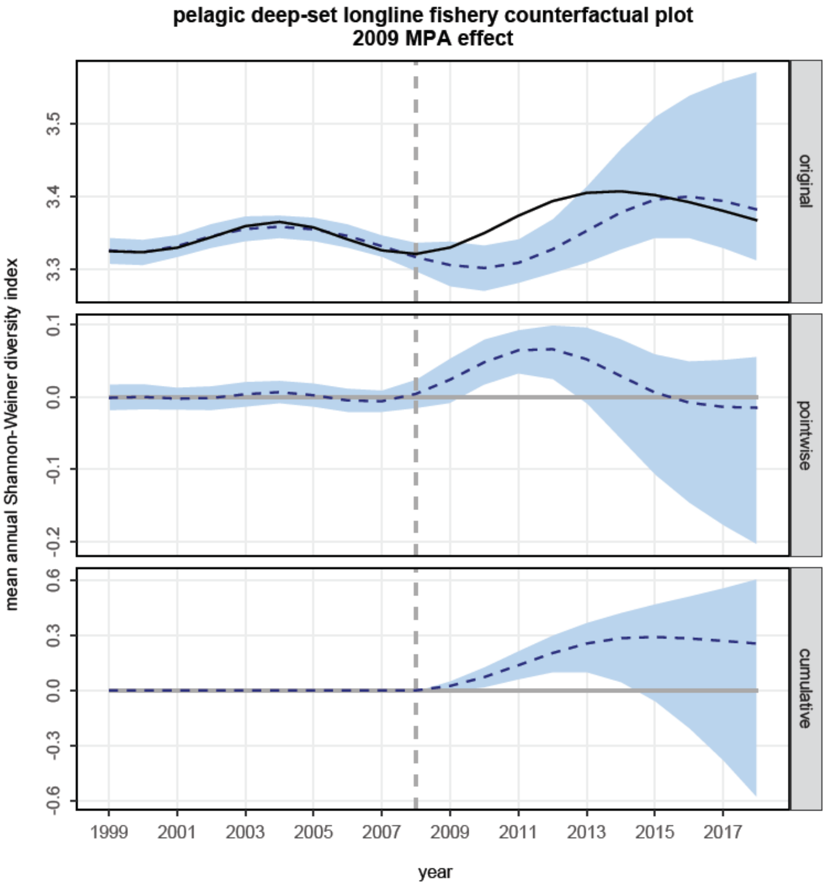

Fig. S9. Counterfactual prediction for annual mean Shannon index H' response to combined MPAs established in 2009 around Johnston Atoll, and Kingman Reef/Palmyra Atoll.

S5. References

Brewer A, Williamson M. A new relationship for rarefaction. Biodivers Conserv. 1994; 3:373–379.

Brooks M, Kristensen K, van Benthem K, ArniMagnusson A, Berg C, Nielsen A, et al. glmmTMB balances speed and flexibility among packages for zero-inflated generalized linear mixed modeling. The R Journal. 2017; 9: 378-400.

Choy C, Wabnitz C, Weijerman M, Woodworth-Jefcoats P, Polovina J. Finding the way to the top: How the consumption of oceanic mid-trophic micronekton groups determines apex predator biomass in the central North Pacific. Mar Ecol Prog Ser. 2016; 549: 9-25.

Executive Office of the President. Proclamation 8031 – Establishment of the Northwestern Hawaiian Islands Marine National Monument. Federal Register. 2006; 71: 36441-36475.

Executive Office of the President. Proclamation 9478- Papahānaumokuākea Marine National Monument Expansion. Federal Register. 2016; 81: 60225-60234.

Fasiolo M. Nedellec R. Goude Y, Wood S. Scalable visualisation methods for modern Generalized Additive Models. arXiv. 2019; https://arxiv.org/abs/1809.10632.

Free C, Thorson J, Pinsky M, Oken K, Wiedenmann J, Jensen O. Impacts of historical warming on marine fisheries production. Science. 2019; 363: 979-983.

Froese R, Pauly D. (Editors). FishBase. 2019; www.fishbase.org. Version 02/2019.

Gelfand A, Schliep E. Spatial statistics and Gaussian processes: a beautiful marriage. Spat Stat. 2016; 18 (Part A): 86-104.

Hastie T, Tibshirani R. Varying-coefficient models. J Roy Stat Soc B. 1993; 55: 757-796.

Heck KL, van Belle G, Simberlof D. Explicit calculation of the rarefaction diversity measurement and the determination of sufficient sample size. Ecology. 1975; 56: 1459–1461.

Hijmans R. geosphere: Spherical Trigonometry. R package version 1.5-10. Comprehensive R Archive Network (CRAN). 2019; https://CRAN.R-project.org/package=geosphere.
Hill M. Diversity and evenness: a unifying notation and consequences. Ecology. 1973; 54:427–432.
Hsie T, Ma K, Chao A. iNEXT: an R package for rarefaction and extrapolation of species diversity (Hill numbers). Methods Ecol Evol. 2016; 7: 1451-1456.
Huang B, Thorne P, Banzon V, Boyer T, Chepurin G, Lawrimore J, et al. Extended Reconstructed Sea Surface Temperature, Version 5 (ERSSTv5): upgrades, validations, and intercomparisons. J Climate. 2017; 30: 8179–8205.
Kammann E, Wand M. Geoadditive models. Appl Stat. 2003; 52: 1-18.
Kristensen K, Nielsen A, Berg C, Skaug H, Bell B. TMB: automatic differentiation and Laplace approximation. J Stat Softw. 2016; 70: 1-21.
Lawton JH, Bignell DE, Bolton B, Bloemers GF, Eggleton P, Hammond PM, et al. Biodiversity inventories, indicator taxa and effects of habitat modification in tropical forest. Nature. 1998; 391: 72–76.
Lüdecke D. ggeffects: Tidy data frames of marginal effects from regression models. J Open Source Sotfw. 2018; 3: 772.
Magurran A. Measuring Biological Diversity. Oxford: Blackwell Science; 2004.
Molony B. Summary of the Biology, Ecology and Stock Status of Billfishes in the WCPFC, with a Review of Major Variables Influencing Longline Fishery Performance. WCPFC-SC1-EB-WP-2. Kolonia, Federated States of Micronesia: Western and Central Pacific Fisheries Commission; 2005.
Newman M, Alexander M, Ault T, Cobb K, Deser C, Di Lorenzo E, et al. The Pacific Decadal Oscillation, Revisited. Journal of Climate. 2016; 29: 4399-4427.
NMFS. Fisheries off west coast states and in the western Pacific pelagic fisheries; pelagic longline fishing restrictions, seasonal area closures, limit on swordfish fishing effort, gear restrictions, and other sea turtle take mitigation measures. U.S. National Marine Fisheries Service. Federal Register. 2004; 69: 17329–17354.
NMFS. International Fisheries; Pacific Tuna Fisheries; 2013 Bigeye Tuna Longline Fishery Closure in the Eastern Pacific Ocean. Federal Register. 2013b; 78: 65887-8.
NMFS. International Fisheries; Pacific Tuna Fisheries; 2014 Bigeye Tuna Longline Fishery Closure in the Eastern Pacific Ocean. Federal Register. 2014; 79: 63562-3.
NMFS. Western and Central Pacific Fisheries for Highly Migratory Species; 2015 Bigeye Tuna Longline Fishery Closure. Federal Register. 2015b; 80: 44883-4.
NMFS. International Fisheries; Pacific Tuna Fisheries; 2015 Bigeye Tuna Longline Fishery Closure in the Eastern Pacific Ocean. Federal Register. 2015c; 80: 46515-6.
NMFS. Western and Central Pacific Fisheries for Highly Migratory Species; 2016 Bigeye Tuna Longline Fishery Closure. Federal Register. 2016a; 81: 45982-3.
NMFS. International Fisheries; Pacific Tuna Fisheries; 2016 Bigeye Tuna Longline Fishery Closure in the Eastern Pacific Ocean. Federal Register. 2016b; 81: 46614-5.
NMFS. International Fisheries; Pacific Tuna Fisheries; 2016 Bigeye Tuna Longline Fishery Reopening in the Eastern Pacific Ocean. Federal Register. 2016c; 81: 69717-8.
NMFS. Western and Central Pacific Fisheries for Highly Migratory Species; 2017 Bigeye Tuna Longline Fishery Closure. Federal Register. 2017a; 82: 37824-5.
NMFS. International Fisheries; Pacific Tuna Fisheries; 2017 Bigeye Tuna Longline Fishery Closure in the Eastern Pacific Ocean. Federal Register. 2017b; 82: 41562-3.
NMFS. Hawaii Longline Observer Program Field Manual. Manual Version LM.17.02. Honolulu: Pacific Islands Regional Office, National Marine Fisheries Service; 2017c.
NMFS. Pacific Island Pelagic Fisheries; False Killer Whale Take Reduction Plan; Closure of Southern Exclusion Zone. Federal Register. 2018; 83: 33848-33851.
NOAA. Multivariate ENSO Index Version 2 (MEI.v2). Available online, www.esrl.noaa.gov/psd/enso/mei/, accessed 2 Jan. 2020. Boulder, USA: Physical Sciences Division, ESRL, NOAA; 2020.
Okamoto H, Bayliff W. A review of the Japanese longline fishery for tunas and billfishes in the eastern Pacific Ocean, 1993-1997. Inter-American Tropical Tuna Commission Bulletin. 2003; 22: 221-431.
Oksanen J, Blanchet F, Friendly M, Kindt R, Legendre P, McGlinn D, et al. vegan: Community Ecology Package. R package version 2.5-6. Comprehensive R Archive Network (CRAN). 2019; https://CRAN.R-project.org/package=vegan.

Pante E, Simon-Bouhet B. marmap: A package for importing, plotting and analyzing bathymetric and topographic data in R. PLoS One. 2013; 8: e73051.
Rigby R, Stasinopoulos D. Generalized additive models for location, scale and shape (with discussion). Appl Stat. 2005; 54: 507-554.
Shannon C. A mathematical theory of communication. Bell System Technical Journal. 1948; 27: 379–423.
Shannon L, Coll M, Bundyu A, Gascuel D, Heymans J, Kleisner K, et al. Trophic level-based indicators to track fishing impacts across marine ecosystems. Mar Ecol Prog Ser. 2014; 512: 115-140.
Smith M, Zhang J, Coleman F. Effectiveness of marine reserves for large-scale fisheries management. Can J Fish Aquat Sci. 2006; 63: 153-164.
Thorley J, Mantua N, Hare S. rpdo: Pacific Decadal Oscillation index data. R package version 0.2.5. The 2018; Comprehensive R Archive Network (CRAN). https://CRAN.R-project.org/package=rpdo.
Tveteras S, Asche F, Bellemare M, Smith M, Guttormsen A, Lem A, et al. Fish Is Food - The FAO's Fish Price Index. PLoS ONE. 2012; 7: e36731.
Wickham H. ggplot2: Elegant Graphics for Data Analysis. 2nd Edition. New York: Springer-Verlag; 2016.
Wilke C. cowplot: Streamlined plot theme and plot annotations for "ggplot2" R package version 1.0.0. 2019; The Comprehensive R Archive Network (CRAN): https://CRAN.R-project.org/package=cowplot.
Wood S. Generalized Additive Models: an introduction with R. Boca Raton: Chapman and Hall/CRC; 2006.
Zeileis A, Fisher J, Hornik K, Ihaka R, McWhite C, Murrell P, et al. Colorspace: a toolbox for manipulating and assessing colors and palettes. arXiv. 2019; 1903.06490.v1. http://arxiv.org/abs/1903.06490.
Zhang T, Andrew Hoell A, Perlwitz J, Eischeid J, Murray D, et al. Towards probabilistic multivariate ENSO monitoring. Geophys Res Lett. 2019; 46: 10532-10540.
