# Supplementary material for: Correction: Ecological responses to blue water MPAs
Source: PLoS One. 2020 Aug 27;15(8):e0238558. doi: 10.1371/journal.pone.0238558 (PMC7451526; doi:10.1371/journal.pone.0238558)
Supplement: S2 File — (PDF) [file pone.0238558.s002.pdf]

PLOS ONE

RESEARCH ARTICLE

Ecological responses to blue water MPAs

Eric Gilman[1,2☯]*, **Milani Chaloupka**[3☯], **Mark Fitchett**[4], **Danielle L. Cantrell**[5], **Matt Merrifield**[2]

1 Pelagic Ecosystems Research Group, Honolulu, Hawaii, United States of America, **2** The Nature Conservancy, Honolulu, Hawaii, United States of America, **3** Ecological Modelling Services Pty Ltd & Marine Spatial Ecology Lab, University of Queensland, Brisbane, Australia, **4** Western Pacific Regional Fishery Management Council, Honolulu, Hawaii, United States of America, **5** US Fish and Wildlife Service, Pacific Remote Islands Marine National Monument, Honolulu, Hawaii, United States of America

☯ These authors contributed equally to this work.
* FisheriesResearchGroup@gmail.com

Check for updates

OPEN ACCESS

Citation: Gilman E, Chaloupka M, Fitchett M, Cantrell DL, Merrifield M (2020) Ecological responses to blue water MPAs. PLoS ONE 15(7): e0235129. https://doi.org/10.1371/journal.pone.0235129

Editor: Heather M. Patterson, Department of Agriculture, Water and the Environment, AUSTRALIA

Received: March 4, 2020

Accepted: June 8, 2020

Published: July 8, 2020

Copyright: This is an open access article, free of all copyright, and may be freely reproduced, distributed, transmitted, modified, built upon, or otherwise used by anyone for any lawful purpose. The work is made available under the Creative Commons CC0 public domain dedication.

Data Availability Statement: The fisheries observer program data used in this study, which contain confidential business information, are owned by and are available from the U.S. government agency NOAA Fisheries https://inport.nmfs.noaa.gov/inport/item/21854 but restrictions apply to their availability. Under the terms of a non-disclosure agreement with U.S. NOAA that the authors who analyzed the observer data had to execute, and under sections 1905 and 201-209 of Title 18 of the United States Code (referred to as the Trade Secrets Laws and Conflict of Interests

Abstract

Marine protected areas (MPAs) can contribute to protecting biodiversity and managing ocean activities, including fishing. There is, however, limited evidence of ecological responses to blue water MPAs. We conducted the first comprehensive evaluation of impacts on fisheries production and ecological responses to pelagic MPAs of the Pacific Remote Islands Marine National Monument. A Bayesian time series-based counterfactual modelling approach using fishery-dependent data was used to compare the temporal response in the MPAs to three reference regions for standardized catch rates, lengths, trophic level of the catch and species diversity. Catch rates of bigeye tuna, the main target species (Kingman/Palmyra MPA, causal effect probability >99% of an 84% reduction; 95% credible interval: -143%, -25%), and blue shark (Johnston MPAs, causal effect probability >95%) were significantly lower and longnose lancetfish significantly higher (Johnston MPAs, causal effect probability >95%) than predicted had the MPAs not been established, possibly from closing areas near shallow features, which aggregate pelagic predators, and from 'fishing-the-line'. There were no apparent causal impacts of the MPAs on species diversity, lengths and trophic level of the catch, perhaps because the MPAs were young, were too small, did not contain critical habitat for specific life-history stages, had been lightly exploited or experienced fishing-the-line. We also assessed model-standardized catch rates for species of conservation concern and mean trophic level of the catch within and outside of MPAs. Displaced effort produced multi-species conflicts: MPAs protect bycatch hotspots and hotspots of bycatch-to-target catch ratios for some at-risk species, but coldspots for others. Mean trophic level of the catch was significantly higher around MPAs, likely due to the aggregating effect of the shallow features and there having been light fishing pressure within MPAs. These findings demonstrate how exploring a wide range of ecological responses supports evidence-based evaluations of blue water MPAs.

Laws, respectively), the authors are prevented from making the U.S. government observer program data publicly available. All other datasets used in this study are open access and citations to these datasets are included in the article.

Funding: This work received funding from The Nature Conservancy http://nature.org to EG and from the Western Pacific Regional Fishery Management Council http://www.wpcouncil.org/ to MC. The funders had no role in study design, data collection and analysis, decision to publish, or preparation of the manuscript. EG received funding support for salary from The Nature Conservancy and MC received funding support for salary from the Western Pacific Regional Fishery Management Council. Author MF, who is an employee of the Western Pacific Regional Fishery Management Council, contributed to the study design and preparation of the manuscript, as explained in the Author Roles statement. Author MM, who is an employee of The Nature Conservancy, contributed to preparation of the manuscript, as explained in the Author Roles statement. Identified in the author affiliations, some of the authors work for private companies, while others work for government agencies or environmental non-governmental organizations.

Competing interests: All authors are involved in fisheries management or provide fisheries advice in ways that can be perceived as competing interests. Some of the authors are employed by national fisheries agencies or work for environmental non-governmental organizations that advocate for specific fisheries policies. The academic scientists have received funding in the past from various sources, including government fisheries agencies, seafood companies and environmental non-governmental organizations. Some of the authors work for private companies, identified in the author affiliations. The authors' affiliations, including with private companies, government agencies and environmental nongovernmental organizations, did not alter the authors' compliance with PLoS ONE policies on sharing data and materials.

Introduction

Marine protected areas (MPAs) and other spatial management measures are increasingly used as components of management frameworks to govern marine activities, including fishing, and to protect the component manifestations of marine biodiversity. A large body of evidence has demonstrated the ecological changes that occur within and adjacent to coastal, benthic, shallow-water MPAs and MPA networks that reduce fishing mortality. These ecological responses include, on average, increased: local abundance and biomass, mean lengths, recruitment and absolute biomass, and species richness and diversity [1–6]. These ecological effects are strongest for upper trophic level taxa with certain behavioral and life-history traits, that have high site fidelity and relatively limited mobility, and that were highly exploited when the MPA was established [7–11]. The magnitude of responses is also higher the larger the magnitude of pressures that were reduced within the MPA, the larger and older the MPA, and the more robust the management system and concomitant higher compliance with MPA rules that reduce pressures [1, 12–14].

Over the past decade there has been a proliferation of very large MPAs that include or are exclusively pelagic or 'blue water' habitat, in which pelagic fishing is restricted or prohibited [15]. These pelagic MPAs contribute substantially towards meeting the area-based goal of Aichi Biodiversity Target 11 and Sustainable Development Goal target 14.5 [16–17]. However, there is extremely limited theoretical—and in particular empirical—evidence of ecological responses to pelagic MPAs and other spatial management approaches for pelagic fisheries [18–20]. Studies that provide a robust basis for inferring causation of ecological as well as socio-economic impacts of pelagic MPAs using counterfactual-based modeling is a priority [20–22].

This limited body of research leaves a substantial gap in understanding how pelagic MPAs can contribute to the management and mitigation of pressures from pelagic fisheries. Direct fishing mortality by pelagic marine fisheries is the main driver of reductions in the size and abundance of pelagic apex predators, including of target stocks and incidentally caught species, although there has been disagreement over the magnitude of these declines [23–27]. Fisheries that target tuna and tuna-like species (Scombroidei), billfishes (Xiphioidei) and other relatively fecund species can have large impacts on incidentally caught species that, due to their lower reproduction rates and other 'slow' life-history traits, are relatively vulnerable to increased mortality, including seabirds, sea turtles, marine mammals, elasmobranchs and some teleosts [28–30]. Pelagic fisheries selectively remove individuals based on certain traits (e.g., behavioral traits for boldness; life-history traits for size-at-age; physiological traits for visual acuity; morphological traits for mouth dimensions), reducing intraspecific genetic diversity and fitness, altering evolutionary processes [31–32]. Fishing gear can alter and damage habitat (e.g., drifting fish aggregating devices or FADs can alter the natural behavior and ecology of species that associate with the gear; derelict FADs can damage sensitive coastal habitats; [33–35]). Fishing mortality of large, highly migratory pelagic predators of high trophic levels (TL> 4.0) can modify pelagic ecosystems' structure and processes, manifested through top-down food web linkages, where these changes can be protracted or permanent regime shifts [27, 36–38]. At this latter broad level, there is limited understanding of what magnitudes of interacting natural (e.g., large-scale climate variability) and anthropogenic pressures (including from fishing) cause pelagic ecosystems to undergo regime shifts [36, 39–41]). Pressures from marine capture fisheries interact with pressures from the four other main drivers of change and loss of marine biodiversity of climate change, marine pollution, habitat degradation and the spread of invasive alien species [42–43].

There is substantial uncertainty over the feasibility of pelagic MPAs to contribute to managing these pressures and achieving ecological objectives. Objectives may include maintaining

targeted biomass levels of highly migratory species, reducing the magnitude of fisheries-induced evolution and maintaining pelagic ecosystems in a quasi-stable state selected to sustain the provision of desired services, including fishery yields [18, 20, 44].

We conducted the first comprehensive performance assessment of very large, pelagic MPAs that banned commercial fishing, established by the United States government at Palmyra Atoll, Kingman Reef and Johnston Atoll, which are components of the Pacific Remote Islands Marine National Monument. The study employed a Bayesian time series-based counterfactual modelling approach using a unique 24.7-year dataset of fishery-dependent observer data from Hawaii's tuna longline fishery to compare the temporal response in the MPAs to three control zones for standardized catch rates, pelagic ecosystem state indicators of community structure (mean length and mean trophic level of the catch, TLc), and species diversity using the the Shannon-Wiener Index. We also assessed model-standardized catch rates for species of conservation concern, species diversity and mean trophic level of the catch within and outside of the MPAs. The assessment of standardized catch rates for species of conservation concern determined if the MPAs have been protecting a bycatch hotspot, or otherwise have not affected or exacerbated bycatch of some at-risk taxa through displaced effort. The comparison of the Shannon diversity index and mean TLc between areas closed vs. open to fishing enabled assessment of the effect of displaced effort on species-level biodiversity and relative degree of disturbance to community structure. Findings augment a small body of theoretical and observed evidence of ecological responses to pelagic MPAs, in particular from studies that provide a strong basis for causal inference [20], improving the knowledge of optimal designs for pelagic MPAs to achieve ecological objectives.

Results

Standardized catch rates

To exemplify the approach employed to develop standardized catch time series, Supporting Information S4-S6 Figs in S1 File. present outcomes of the Gaussian spatially-explicit generalized additive mixed model (geoGAMM) for the bigeye tuna (*Thunnus obesus*) standardized catch rate for the full study period, before and after Hawaii's longline fishery was exposed to the MPAs. S4 Fig in S1 File. identifies 12 significant model terms included in the geoGAMM for the bigeye tuna standardized catch rate. S5 Fig in S1 File. shows the residual spatial effect of the geoGAMM, identifying locations with high unexplained bigeye tuna catch rates (i.e., areas with high bigeye tuna catch rates that were weakly explained by the model). And, S6 Fig in S1 File. presents the temporal trends in standardized bigeye tuna catch rates estimated by the geoGAMM for each study zone (three control zones, three treatment zones).

Fig 1 presents the counterfactual catch rate with standardized effort (relative abundance) prediction for the 110 nm treatment zone at Kingman Reef/Palmyra Atoll (hereafter referred to as KP110) for bigeye tuna. In the top panel, the solid line is the "observed" (predicted from the geoGAMM) relative abundance response, and the curved dashed line is the Bayesian state-space structural time series model fit to generate the counterfactual relative abundance prediction (i.e., what relative abundance would have been had the MPA not been established). The bottom panel is the pointwise difference between the two curves of the first panel, demonstrating the temporal dynamics of the apparent response to the MPA intervention. In the top panel, the dashed line to the left of the vertical line is the modeled prediction, and to the right of the vertical line is the counterfactual prediction post-intervention (i.e., what the response would have been in the treatment zone had there not been an intervention). The vertical dashed line indicates the beginning of the counterfactual prediction period (the end of

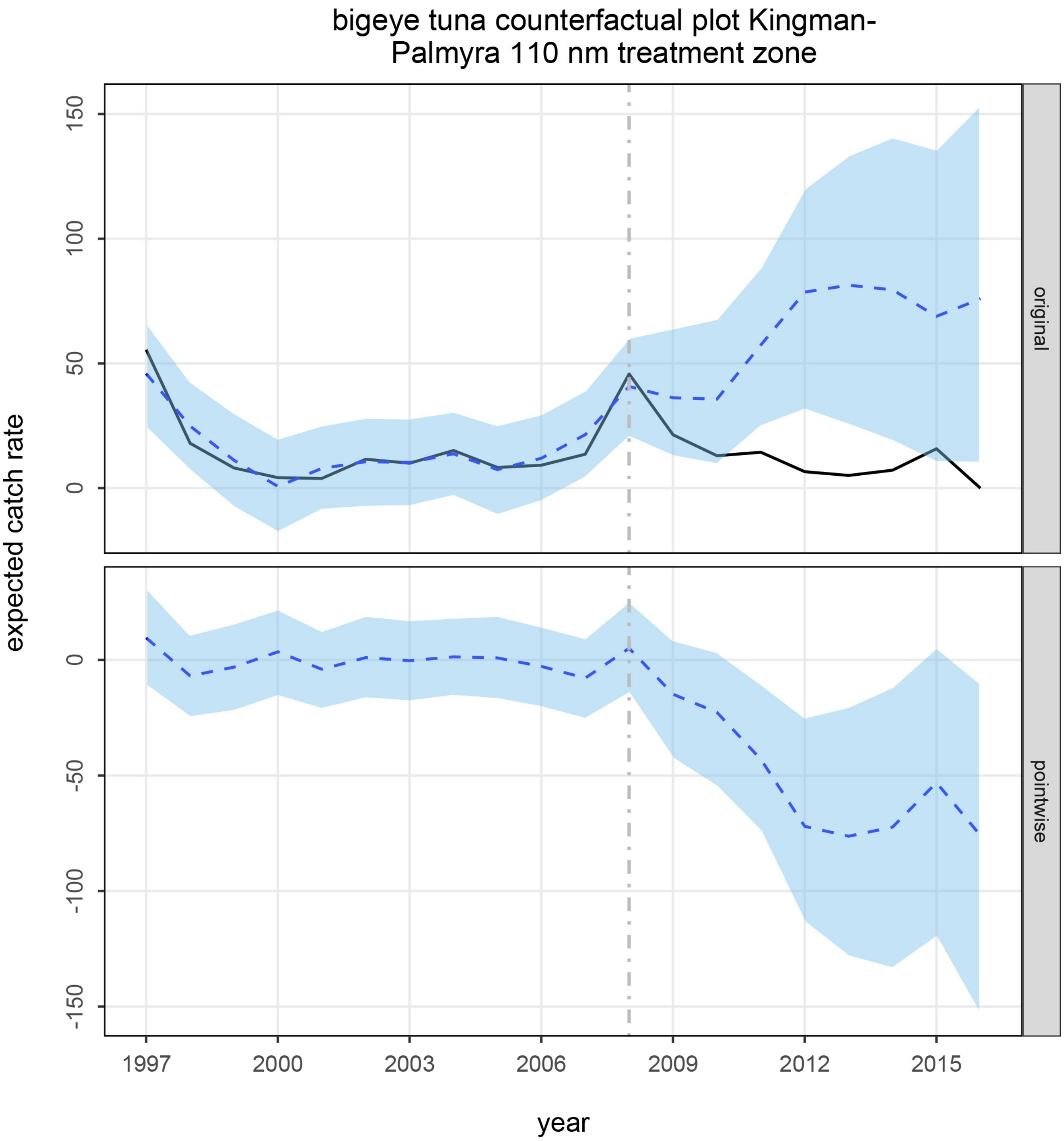

Fig 1. Counterfactual prediction plot for the causal impact of a 2009 intervention on bigeye tuna catch rates within a 110 nm zone around the Kingman Reef and Palmyra Atoll MPA. Top panel: Solid curve is the expected catch rate for the MPA 110 nm treatment zone from 1997–2016. The dashed curve and polygon show the counterfactual prediction (and prediction uncertainty) drawn from 50k stochastic realizations of a Bayesian state-space structural time series model fitted to the expected reference zone-specific catch rates with other potentially informative predictors. **Bottom panel:** Shows the pointwise difference (and 95% credible interval) between the 2 curves in the top panel (expected catch rate, counterfactual prediction), which shows a significant decline of the expected catch rate following the 2009 intervention event. This reveals the temporal dynamics of the causal impact of the intervention. The posterior probability of a causal effect attributable to the intervention was > 99% of an 84% gradual permanent reduction since 2009 (95% credible interval: -143% to -25%).

https://doi.org/10.1371/journal.pone.0235129.g001

calendar year 2008 for the 50nm MPA established in Jan. 2009). Shaded areas are 95% credible intervals of the counterfactual prediction.

There was a causal effect of a decline in bigeye tuna standardized catch rate attributable to the 2009 Kingman/Palmyra MPA (Fig 1 and Table 1). The impact was large, gradual and permanent. The expected bigeye tuna standardized catch rate within KP110 would have been significantly higher without the establishment of the MPA. Significant causal effects were also observed for standardized catch rates for blue shark (*Prionace glauca*) and for longnose lancetfish (*Alepisaurus ferox*) in both the 110 nm and 260 nm treatment zones at Johnston Atoll (hereafter referred to as J110 and J260, respectively) (Table 1). The expected blue shark and longnose lancetfish standardized catch rates within the treatment zones around Johnston would have been significantly higher and lower, respectively, without establishment of the MPAs (Table 1). The blue shark impacts from the 50 nm and 200 nm MPAs at Johnston were moderate, gradual and permanent for both interventions. The lancetfish impacts from the 50 nm and 200 nm MPAs at Johnston were large and moderate, respectively, and were gradual and permanent for both interventions. There was no compelling evidence for any causal impact on the zone-specific catch rates for other species, nor for bigeye tuna for the MPAs at Johnston, blue shark for the Kingman/Palmyra 50 nm MPA, and for longnose lancetfish for the Kingman/Palmyra 50 nm MPA (each with Bayesian predictive $p > 0.10$).

Length and TLc

There were no causal effects on bigeye or yellowfin tuna (*T. albacares*) mean lengths attributable to the 50 nm MPA within KP110 or to the two MPAs within J260 (Bayesian predictive $p > 0.05$; S7 Fig in S1 File). There were also no causal effects on mean TLc attributable to the 50 nm MPA within KP110 or to the two MPAs within J260 (Bayesian predictive $p > 0.05$; S8 Fig in S1 File).

Fig 2 presents the time series of estimated annual mean TLc for sets made within vs. outside of treatment zones from a GAMM with Gaussian likelihood. The overall mean annual TLc on average was significantly higher within than outside of the treatment zones ($p<0.001$). For each individual year in the time series, mean TLc was higher but 95% uncertainty intervals (UIs) overlap.

ETP catch rates

Fig 3 presents expected mean catch rates (catch per set) and 95% UIs from GAMMs with negative binomial likelihood within future MPAs (i.e., within 50 nm of Kingman/Palmyra, and within 200 nm of Johnston, prior to MPA establishment) vs. outside of MPAs for five species

Table 1. Posterior inference summary for the post-intervention period (2009 onwards) for species-specific catch rate predictions deemed here to indicate a significant MPA attributable causal impact.

zone/species	causal effect probability	relative effect (%)		important synthetic control predictors[1]
		mean	95% credible interval	
**kp110 nm**				
bigeye tuna	> 0.99	-84	(-143, -25)	FAO Fish Price Index, PDO (40%), northwest reference zone (25%)
**j110 nm**				
blue shark	> 0.97	-40	(-83, -0.7)	MEI, southern reference zone
longnose lancetfish	> 0.95	187	(-13, 457)	MEI
**j260 nm**				
blue shark	> 0.96	-29	(-62, 3.8)	southern reference zone
longnose lancetfish	> 0.99	43	(27, 65)	MEI

[1] Unless stated the synthetic control predictor inclusion probability was > 50%. PDO = Pacific Decadal Oscillation, MEI = Multivariate ENSO Index.

https://doi.org/10.1371/journal.pone.0235129.t001

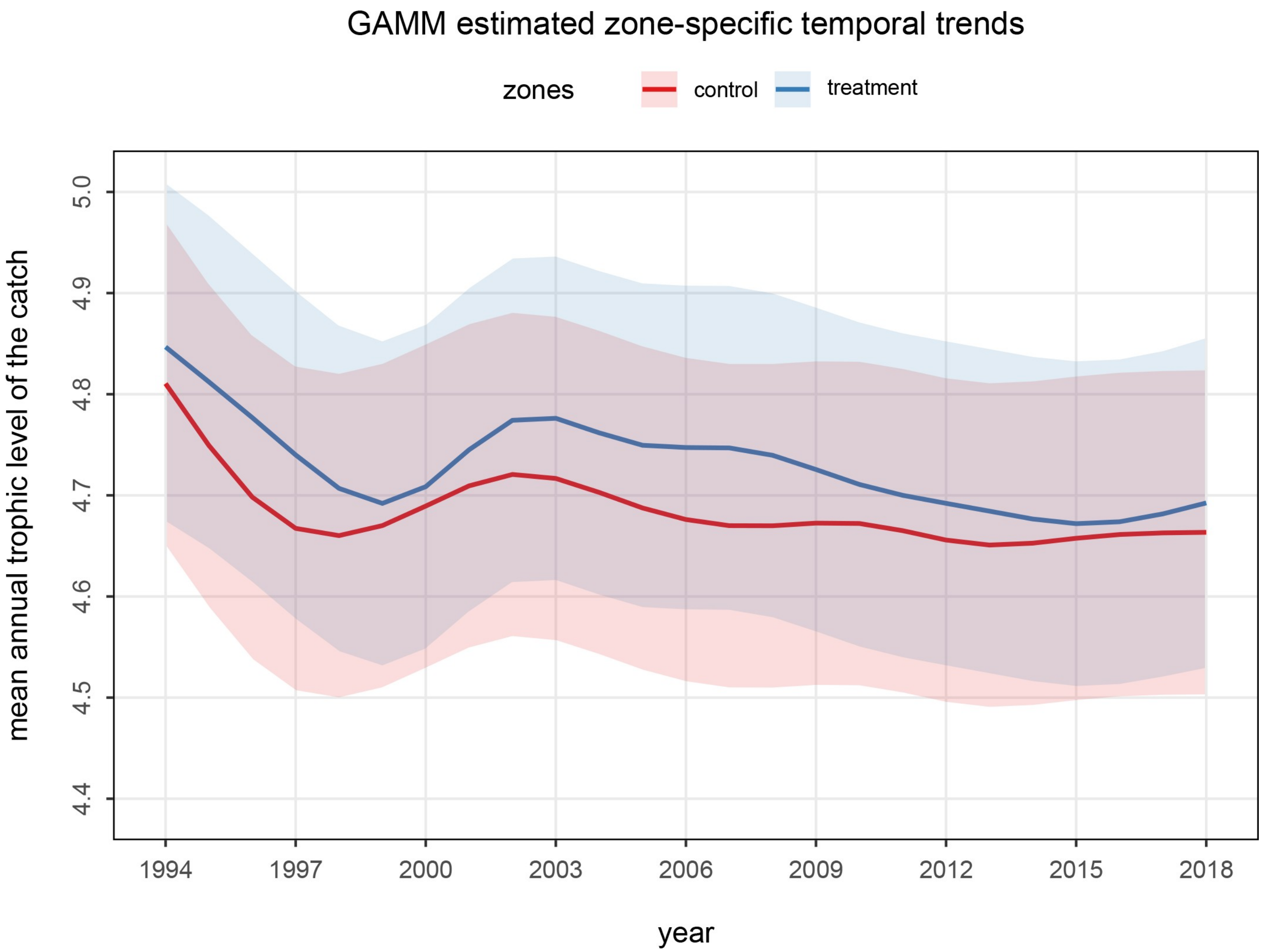

Fig 2. Comparison of the estimated annual trends in the mean trophic level of the catch (TLc) inside (*treatment*) and outside (*control*) combined treatment zones (Kingman/Palmyra 110 nm + Johnston 260 nm) derived using a GAMM regression model with Gaussian likelihood fitted to the set-specific catch data. Solid curves show the zone-specific mean trends and the shaded polygons show the zone-specific 95% confidence intervals.

https://doi.org/10.1371/journal.pone.0235129.g002

of conservation concern (also referred to as endangered, threatened and protected or ETP species). Oceanic whitetip (*Carcharhinus longimanus*), silky (*C. falciformis*) and blue shark mean catch rates were significantly higher, and striped marlin (*Kajikia audax*) catch rates significantly lower, within MPAs than in the rest of the Hawaii longline fishing grounds. There was no meaningful difference in bigeye thresher shark (*Alopias superciliosus*) mean catch rates within vs. outside of MPAs.

Fig 4 presents expected mean catch rates (catch per set) and highest posterior density interval from a binomial likelihood coupled with a Bayes-Laplace beta prior within vs. outside of the 50 nm MPA at Kingman/Palmyra and 200 nm MPA at Johnston for species/groups of conservation concern with too small sample sizes for GAMM fits. Albatrosses and shortfin mako shark (*Isurus oxyrinchus*) mean catch rates were significantly lower, and olive Ridley sea turtle (*Lepidochelys olivacea*) catch rate significantly higher, within MPAs than in the rest of the Hawaii longline fishing grounds. There was no meaningful difference in mean catch rates for odontocetes.

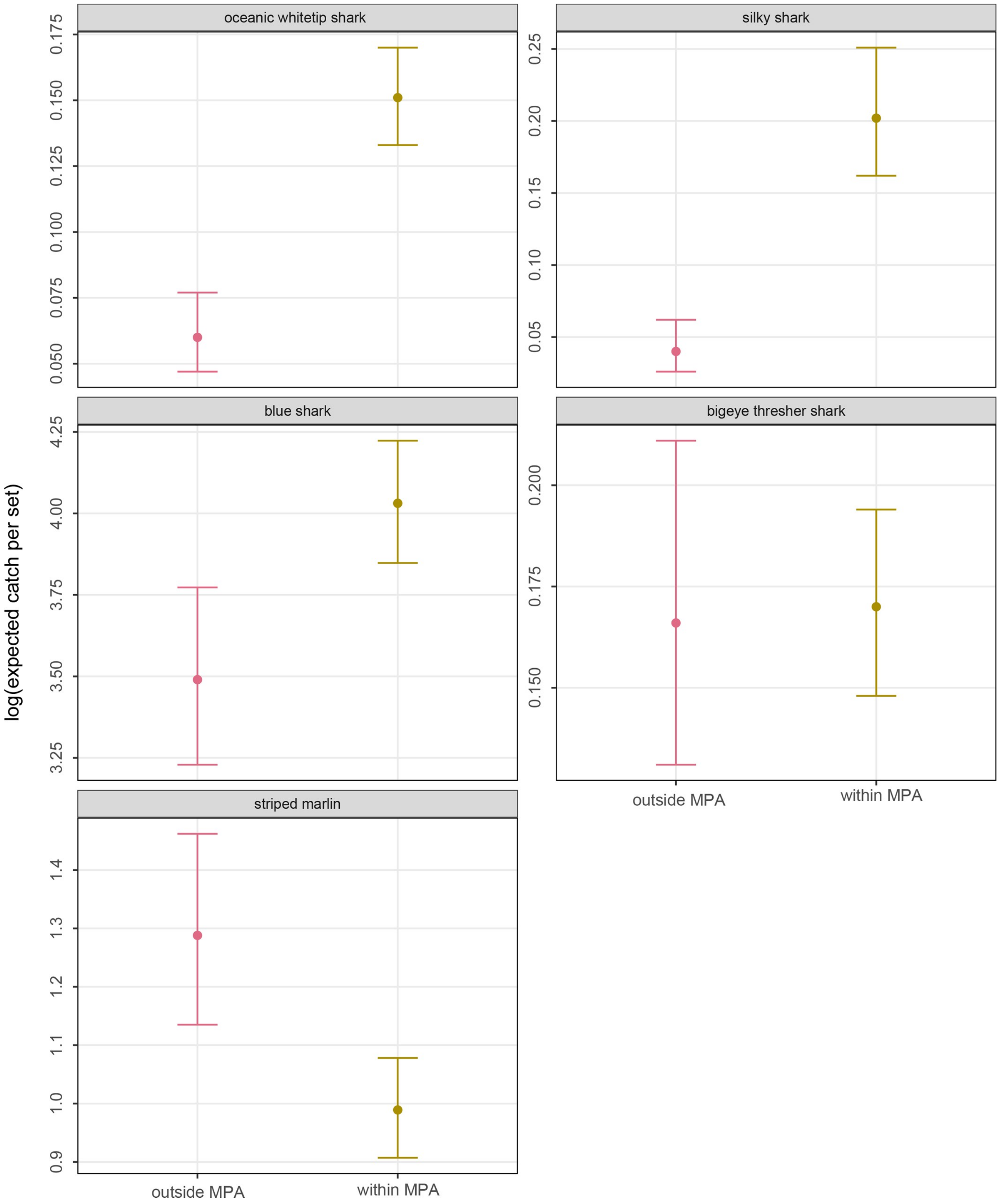

Fig 3. Comparison of the estimated mean catch rates inside and outside of the combined MPAs (Kingman/Palmyra and Johnston) for 5 species of conservation concern derived using species-specific GAMM regression models with negative binomial likelihood fitted to the set-specific catch data. Solid dot shows the expected mean rate and the vertical bar shows the 95% confidence interval.

https://doi.org/10.1371/journal.pone.0235129.g003

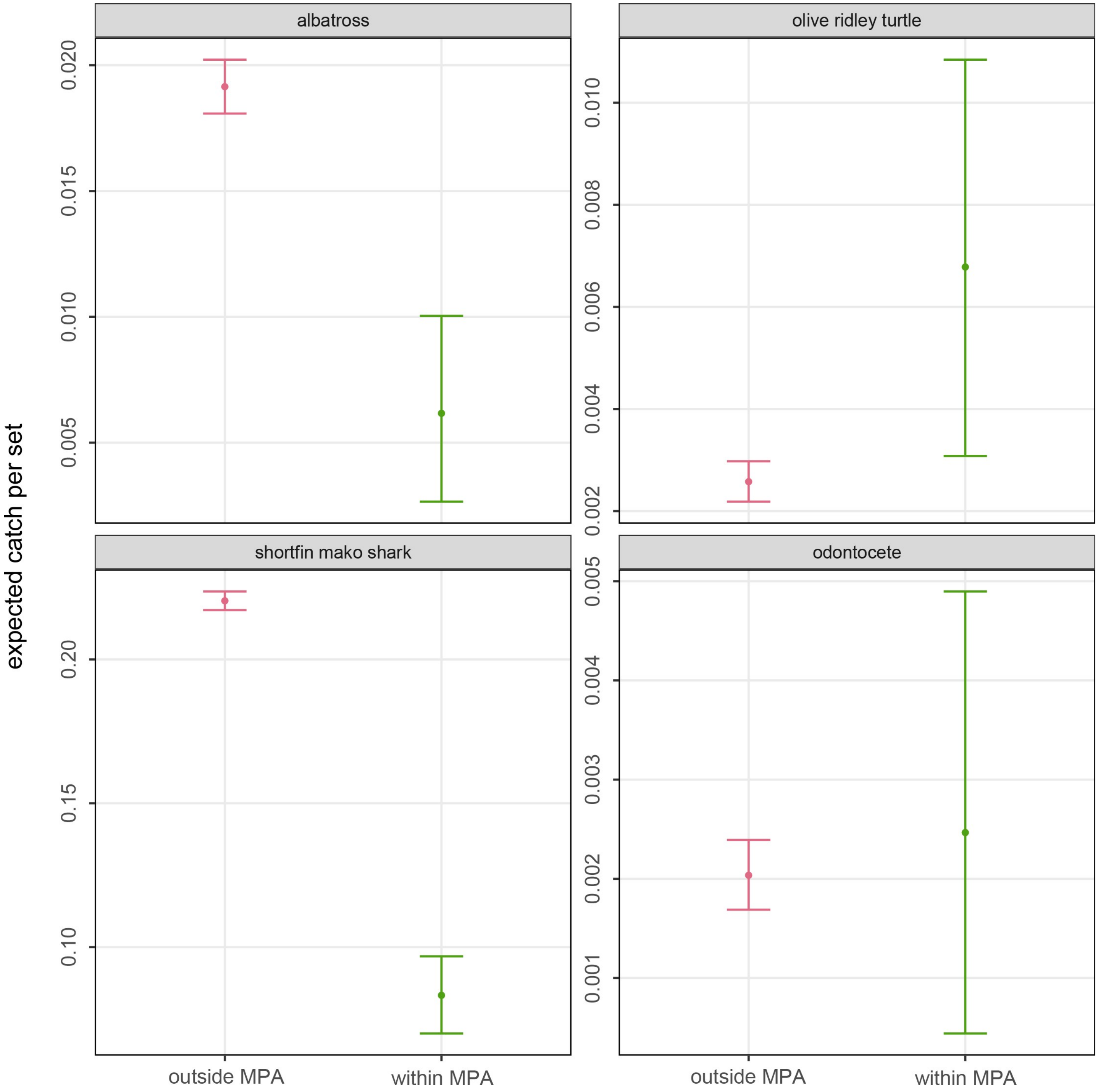

Fig 4. Comparison of the estimated mean catch rates inside and outside the combined MPAs (Kingman/Palmyra and Johnston) for 4 species of conservation concern with only limited catch data. These estimates were derived by sampling the summed catch and effort data from a species-specific binomial likelihood with a noninformative Bayes-Laplace prior. Solid dot shows the posterior mean rate and the vertical bar shows the 95% highest posterior density interval.

https://doi.org/10.1371/journal.pone.0235129.g004

Shannon diversity index

There were no causal effects on the annual mean Shannon index H' attributable to the 50 nm MPAs at Kingman/Palmyra and Johnston (Bayesian predictive $p > 0.05$; S9 Fig in S1 File).

There were no significant differences in mean annual H' within MPAs vs. in the three control zones outside of the MPAs, and there was no trend in H' in any of the four zones. There was major uncertainty in all four estimated trends, and 95% UIs completely overlapped.

Discussion and conclusions

Significant standardized catch rate responses

Within KP110, the significant reduction in standardized catch rate of bigeye tuna, the main target species of the Hawaii deep-set longline fishery, caused by the 50 nm MPA, reduced the economic viability of fishing for the subset of vessels that fish in this already inconvenient, distant part of their fishing grounds. Excluding vessels from areas within treatment zones near atolls/reefs, where the local abundance of some pelagic apex predators is higher than open ocean habitats [45–48], may explain the findings that there would have been significantly higher bigeye tuna (in KP110), higher blue shark (in J110, J260) and lower longnose lancetfish (in J110, J260) standardized catch rates had the MPAs not been established. If correct, then the findings that the MPAs caused a reduction in bigeye tuna and blue shark standardized catch rates do not reflect an ecological response to the MPA of a decline in local abundance of these species within treatment zones. Instead, the reduction in standardized catch rates would have been caused by excluding fishing grounds and displacing effort from areas within the treatment zone with natural aggregating features with relatively high catch rates and local abundance of pelagic apex predators to open ocean areas with relatively lower catch rates and local abundance of these species. There may have been increased local abundance of bigeye tuna, blue shark and other apex predator species susceptible to capture in pelagic longline fisheries in the treatment zones post MPA establishment in response to the MPAs. However, the counterfactual assessment did not detect this response, perhaps because any local abundance response to the MPA was offset by the reduced catch rate from removing the relatively higher productive fishing grounds near the shallow features.

Truncating the fishing grounds within the treatment zones to retain areas with lower relative abundance of apex predators may have resulted in the remaining fishing grounds in the treatment zone having relatively lower predation pressure and competition for prey and higher local abundance of longnose lancetfish and possibly other lower trophic level species [38]. Displacing fishing effort to grounds with lower relative abundance of top pelagic predators may also have resulted in the remaining fishing grounds in the treatment zone having relatively lower gear saturation and interference competition [49–50], contributing to causing the observed longnose lancetfish standardized catch rate to be higher than the counterfactual prediction.

Fishing-the-line (when vessels fish along the MPA boundary) post-MPA establishment could be another partial explanation for these findings. Post MPA establishment, the catch levels of large pelagic species by foreign fishing vessels may have increased along the seaward margins of the US exclusive economic zone (EEZ) adjacent to Kingman/Palmyra and Johnston [51–52]. This could have caused the observed standardized catch rates (i.e., predicted from the geoGAMM) of bigeye tuna and blue shark in the 'spillover' portions of treatment zones to be lower than counterfactual predictions. If fishing mortality levels of bigeye tuna and blue shark in these areas post-2009 were instead the same as pre-2009, then the Hawaii longline standardized catch rate might have increased as predicted by the counterfactual assessment. A post-2009 increase in fishing mortality by foreign vessels of blue shark and possibly other top apex predators in waters near the Johnston MPA may explain why the observed longnose lancetfish standardized catch rate was higher than the counterfactual prediction, for the same reasons hypothesized above of how reduced local abundance of apex predators might cause increased

standardized catch rates of lower trophic level species. It is a research priority to investigate international fishing effort around the Monument's seaward margins.

ETP catch risk within vs. outside of MPAs

The MPAs' displacement of Hawaii longline fishing effort resulted in conflicting effects on bycatch rates of ETP species. The fishing grounds in areas that are now MPAs were bycatch hotspots for some ETP species and coldspots for others. Managers have not evaluated whether these unplanned conflicting effects are acceptable tradeoffs, such as by estimating relative risks or absolute population-level effects.

There have been similar observations of fishery closures designed to reduce the bycatch of one species of conservation concern that displaced fishing effort and inadvertently exacerbated bycatch rates of other at-risk taxa [20, 53–55]. There are also observations of MPAs resulting in the spatial or temporal displacement of fisheries bycatch of an individual species or age classes of a species [20, 56–58].

In fisheries with quotas for target species, such as for Hawaii's tuna longline fishery for bigeye tuna, but no effort controls for individual vessels, MPAs that displace fishing effort to areas or periods with relatively lower target species catch rates could result in increased effort to maintain target species catch levels. This in turn could result in increased catch and fishing mortality levels of bycatch species, including of ETP species (e.g., [18]). This was likely not the case for the MPAs assessed here because bigeye tuna catch rates were similar within and outside of the closed areas. The bycatch hotspots and coldspots within the MPAs were therefore also hot and coldspots when expressed as bycatch-to-target catch ratios (e.g., [59]), at least for this fishery's main target species of bigeye tuna.

TLc within vs. outside of treatment zones

The difference in mean TLc between the treatment and control zones was mainly due to there having been 70% and 45% higher nominal catch rates of relatively higher trophic level species of tunas (main species bigeye tuna, TL = 4.81) and sharks (main species blue shark, TL = 4.95), respectively, and 44% lower catch rates of relatively low trophic level species of other teleosts (main species longnose lancetfish, TL = 4.34) within treatment zones. The observed higher mean TLc near Johnston and Palmyra Atolls and Kingman Reef is likely due to the aggregating effect of shallow submerged features and islands on pelagic predators (and their prey), including sharks [47–48, 60], seabirds [46, 61], sea turtles [48], marine mammals [46, 62], and teleosts, including some tunas and billfishes [48, 63–67].

The higher mean TLc in treatment zones relative to control zones might also be a result of the treatment zones having experienced relatively light effort by fisheries selecting for upper trophic level species. Longline fishery removals of upper trophic level species may not have altered the relative abundance of upper trophic level species near the shallow features possibly because fishing effort was light and because of the short residency times and hence short time for replenishment of these highly migratory apex predators at these sites. The treatment zones might therefore have relatively undisturbed community structures, characteristic of the mean TLc when a fishery expands to new areas [68–69], instead of a lower mean TLc characteristic of areas that have had longer and more extensive exploitation histories during which fishing down or through the food web has occurred [70–71].

The displacement of Hawaii longline effort from the MPAs with higher mean TLc to the rest of the fishing grounds with lower mean TLc, may have diverted fishing from areas with relatively higher to lower local abundance of top trophic level species. This would have contributed a small reduction in pressure on higher trophic level species and small increase in

pressure on lower trophic level species within the broad fishing grounds of Hawaii's fishery. This contributes to more balanced exploitation [72–73] across these higher pelagic trophic levels. However, as the counterfactual model prediction found few or no local, relative abundance response in assessed species, and because a regional, absolute abundance response was unlikely to have been caused by the MPAs [9, 20], a response in ecosystem structure was also unlikely to have resulted from the MPAs.

Shannon diversity index within vs. outside of MPAs

The lack of an observed difference in Shannon diversity index between the MPAs and the rest of the fishing grounds indicates that, with species weighted according to their relative frequency, these areas had similar diversity (richness and evenness) of species susceptible to capture in pelagic longline gear, perhaps because these zones experienced similar degrees of disturbance to species-level diversity. In general, the higher the magnitude or frequency of disturbance, the lower the species diversity, however, the relationship is variable [74–76]. The Shannon index is correlated with several other commonly used species diversity metrics (e.g., [77]), and provides a useful characterization of diversity by not favoring rare or dominant species, as all species are weighted according to their relative frequency. While higher pelagic predator species richness and local abundance of some upper trophic level species occur proximate to shallow seamounts and other submerged features relative to open ocean habitat [45, 47–48], evenness at shallow features relative to open ocean habitat is not well understood.

Non-significant counterfactual predictions of response to MPAs

There were no significant counterfactual predictions of mean length, mean TLc nor Shannon index responses to the MPAs, and no meaningful local abundance responses for most or possibly all assessed species/treatment zone combinations. The lack of meaningful ecological responses to the MPAs for these attributes may have been due to:

- **Size**: The MPAs may be too small to have protected a large enough proportion of populations and to have retained individuals for a sufficient proportion of their lifetime. A pelagic MPA, or network of pelagic MPAs, would need to be extremely large, covering a large proportion of a population's distribution, and protecting a substantially large proportion of the individuals of a population, in order to substantially reduce the risk of fishing mortality of an individual pelagic organism throughout its lifetime [9, 11, 44, 78–79]. While there is limited understanding of spawning behaviors of most pelagic species [80], bigeye, yellowfin, skipjack and albacore tunas are currently understood to have extensive spawning grounds in tropical and subtropical waters and protracted spawning seasons [79, 81]. While tuna spawning habitat very likely occurs within the monument components assessed here and other pelagic MPAs of the tropical Pacific [82], for these highly fecund broadcast spawners, protecting a small proportion of the distribution of spawning stock biomass likely has minimal effect on recruitment or absolute biomass, where only at extremely low population sizes would egg production likely be a limiting factor for recruitment [83,84]. If individuals are transient, remaining for relatively short time periods (days, weeks) in an MPA, especially if fishing-the-line occurs, then there would not be an increase in biomass from the MPA, locally or stock-wide [11, 20, 85–86]. However, at static pelagic sites containing networks of natural and non-natural aggregating features (shallow seamounts, anchored FADs and buoys, banks and ledges), if large pelagic species have sufficiently long persistence (e.g., residency times of months to years [66, 87–88]), MPAs theoretically could provide protection to individuals for

a sufficient proportion of their lifetime during which a large proportion of their total growth occurs, augmenting growth and local biomass within the MPA.

- **Age**: The age of an MPA can significantly explain ecological responses [12, 14, 89]. The counterfactual assessments of the 50 and 200 nm closures employed time series after the treatments of 9- and 4-year durations, respectively. There were even shorter time series of length data. Perhaps in time ecological responses to the MPAs will become apparent.
- **Site selection**: The MPAs may not contain critical habitat for certain life-history stages of these highly migratory species that are susceptible to capture in pelagic longline gear. Pelagic MPAs that protect habitat in locations and during periods that are important for critical life-history stages of pelagic species could cause increased recruitment and absolute biomass of populations [18, 90]. This includes periods and areas used for spawning, mating and calving/pupping, as well as nursery and nesting areas, areas important for foraging, and migratory pathways [20].
- **Degree of exploitation prior to closures**: The MPAs were marginal fishing grounds for the Hawaii fleet, due likely to their large distance from port, with relatively light exploitation prior to the closures and hence a concomitant small release of pressure subsequent to closure. The magnitude (as well as the type) of pressures that were reduced within an MPA can significantly explain ecological responses [1, 14]. The lack of a response in the Shannon diversity index to the MPAs suggests that the level of fishing mortality that had been occurring in the treatment zones prior to the establishment of the MPAs had not suppressed the number and dominance of species that are susceptible to capture in pelagic longline gear. Similarly, the lack of a mean TLc response suggests that, after the establishment of the MPAs, in treatment zones, there was no change in the size structure of the catch. Discussed above, the observation of higher mean TLc in treatment zones relative to control zones suggests that the treatment zones, prior to the MPA establishment, had experienced relatively light effort and light pressure on upper trophic level species. The magnitude of reduced fishing pressure from establishment of the MPAs may have been inadequate to cause an increase in local abundance of upper trophic level species and concomitant increased predation and reduction in local abundance of lower trophic level species that are susceptible to capture in pelagic longline fisheries.
- **Spillover**: If the MPAs caused increased local abundance within the MPAs of species that are susceptible to longline capture, spillover across the MPA seaward margins may have occurred over a smaller spatial scale than assessed here (tens of km). We explored but could not employ smaller 30 nm spillover areas within counterfactual treatment zones due to inadequate sample sizes of fishing effort. Where MPAs have been documented to result in spillover of fished species that increase in local abundance as a result of site-based protection from fishing mortality, the spillover effect was detectable over very small distances (100s of meters) from the MPA boundary [91]. However, it is possible that the spatial extent of a spillover effect could extend over tens to hundreds of km (e.g., see [92–93]).
- **Fishing-the-line:** Discussed above, after MPAs were established, there may have been increased pelagic fishing effort and mortality near the MPAs' seaward margins.

Discussed in the Section *Significant Standardized Catch Rate Responses*, there may have been increased local abundance of some of the apex predator species susceptible to capture in pelagic longline fisheries in the treatment zones in response to the establishment of the MPAs that was offset (or in the case of bigeye tuna and blue shark, was exceeded) by reduced catch rates from removing the relatively higher productive fishing grounds near the shallow features.

Available evidence suggests that illegal fishing within the MPAs was not an important factor affecting ecological responses to the closed areas. We identified nominal illegal fishing by Hawaii longline vessels within the MPAs. Analysis of Automatic Identification System data for 2013 and 2014 found only one illegal fishing day by a pelagic fishing vessel within the 50 nm protected area around Palmyra Atoll [94]. Additional research could be conducted to assess the extent of illegal fishing in the Monument.

Conclusions and research priorities

This study conducted the first comprehensive performance assessment of large, pelagic MPAs established by the Pacific Remote Islands Marine National Monument. Findings, summarized in Table 2, expand an extremely limited body of empirical evidence with a robust basis for inferring causation of ecological responses to pelagic MPAs and other spatial management approaches for pelagic fisheries [20].

If MPAs are to successfully contribute to meeting objectives of fisheries management, they likely need to be one component of a suite of management tools [100], and need to account for fishers' responses to the MPA, including from effort displacement, and, in response to reduced seafood supply from the MPA, increased effort by other fisheries, which can exacerbate or introduce new adverse ecological consequences [57, 95–97]. As with static habitats, dynamic but persistent habitats are relatively predicable, enabling dynamic pelagic MPA boundaries to be feasibly defined to achieve some ecological objectives (e.g., manage bycatch, [20, 97–98]). But, as with static pelagic MPAs, spatial management of dynamic pelagic habitat would require extremely large areas to achieve other ecological objectives, including maintaining or increasing the absolute abundance of a population of a highly migratory pelagic predator [9, 11, 44, 78–79]. This is particularly true if they are not used in combination with other management measures [99]. The large size of pelagic MPAs necessary to achieve ecological objectives of increased local and absolute biomass, especially if they occur in areas beyond national

Table 2. Summary of key findings and interpretations.

Study Component	Key Findings	Hypothesized Interpretations
**Ecological Responses to the MPAs**		
Standardized catch rates	• MPAs caused significant reductions in bigeye tuna and blue shark standardized catch rates	• MPAs eliminated fishing grounds near shallow submerged features within treatment zones where local abundance of apex predators, including bigeye tuna and blue shark, is relatively high; and pressure, gear saturation and interference competition for lower trophic level species, including longnose lancetfish, is relatively high
	• MPAs caused a significant increase in longnose lancetfish standardized catch rate	
		• Fishing-the-line reduced local biomass of top predators
	• MPAs had no causal impact on standardized catch rates of other assessed species	• MPAs too young
		• MPAs too small
Mean lengths	• MPAs had no causal impact	• MPAs do not contain critical habitat for specific life-history stages
Mean trophic level of the catch	• MPAs had no causal impact	• MPAs had experienced light fishing pressure prior to fishery closure
Species diversity	• MPAs had no causal impact	• Fishing-the-line occurred
**Comparison of Ecological Attributes Between MPAs and Control Zones**		
Standardized catch rates for species of conservation concern	• MPAs protect bycatch hotspots and hotspots of bycatch-to-target catch ratios for some at-risk species, but coldspots for others	• Aggregating effect of shallow submerged features on some pelagic predators
		• Uneven spatial distribution of relative abundance within a species' range
Mean trophic level of the catch	• Significantly higher around MPAs	• Aggregating effect of shallow submerged features
		• MPAs had experienced light fishing pressure prior to fishery closure
Species diversity	• No significant difference in Shannon diversity index between the MPAs and rest of the fishing grounds	• With species weighted according to their relative frequency, these areas had similar diversity of species susceptible to capture in pelagic longline gear

https://doi.org/10.1371/journal.pone.0235129.t002

jurisdiction, makes establishment, surveillance and enforcement extremely challenging [20]. In fisheries where conventional management methods have failed, the underlying causes for failure (management measures do not follow scientific advice, lack of compliance, overcapacity, high levels of illegal fishing, etc.) may also prevent MPAs from meeting objectives [12, 18, 100–101]. When MPA site selection and design is opportunistic to meet political commitments such as the area-based targets, there is a large risk of not achieving biodiversity and fisheries management objectives [18, 102–105].

The study assessed a subset of possible meaningful ecological responses to the MPAs. The study's assessment of ecological responses was limited by using only fishery-dependent data for species susceptible to capture in pelagic longline gear, using non-randomized and non-systematic preferential sampling, a research approach commonly employed in many disciplines (e.g., species distribution modeling, [106]). Evaluating conservation interventions using fishery-dependent data is challenging and can lead to ambiguous conclusions. Finding few significant statistical effects does not mean there was no impact—it is just that we could not find any strong evidence of impacts for some ecological responses given the available fishery-dependent data. Absence of evidence is not evidence of absence. We suspect that impact evaluations of MPAs based on such preferentially-sampled observational data will often be inconclusive and uncertain because of the complex social, economic and ecological interactions affecting pelagic fishery catch rates. The study did not assess various ecological responses to the MPAs that could be explored using both fisheries-independent and fisheries-dependent data, such as changes in attributes (e.g., biomass, size) of non-harvested species, changes from cascading effects through the food web (e.g., effects on seabird populations from changes in prey availability, from subsurface predators driving forage species to the sea surface, and in prey local abundance, in response to reduced localized fishing mortality of apex predators, [107–110], or changes in functional links between open ocean and coastal pelagic, demersal and benthic systems [111]. The study evaluated a small subset of ecological attributes of a wide range of possible variables that might be included as criteria for MPA site selection (e.g., endemic species richness, total species richness and diversity, rarity, [110–114]) and as part of a core set of ecosystem indicators for ecosystem-based fisheries management [115–116]. This performance assessment was therefore not exhaustive. Exploring a wider range of ecological attributes is a priority to support evidence-based evaluation of blue water MPAs.

Due to the larger sample size plus the number of independent studies, correctly designed meta-analyses can provide estimates with increased precision and accuracy over estimates from single studies, with increased statistical power to detect a real effect [117–118]. As has been conducted for coastal, shallow-habitat no-take MPAs [1, 4, 119], once a sufficient number of evidence-based evaluations of both static and dynamic blue water MPAs exist, a global meta-analysis will enable answering unresolved questions over effective designs and performance efficacy in contributing to objectives of fisheries management and biodiversity conservation that are relevant over diverse settings.

Methods

Hypotheses

The study tested six hypotheses:

- The MPAs caused an increase in the local biomass (number of individuals, mean length) of species susceptible to capture in pelagic longline gear within MPA and spillover areas.

- Due to released fishing pressure of apex predators within the MPAs, there was a shift in the size structure of the community, increasing the mean TLc and species-specific mean lengths within MPA and spillover areas.
- The MPAs caused increases in species richness and evenness, as indicated by the Shannon diversity index, within MPA and spillover areas.
- The MPAs have higher species-level biological diversity than the rest of the fishing grounds of Hawaii's tuna longline fishery.
- There were higher catch rates of species of conservation concern in areas that later become MPAs than the rest of the fishing grounds of Hawaii's tuna longline fishery.
- There was higher mean TLc within MPA and spillover areas.

Study period and area

The study period was from 4 March 1994 to 31 October 2018. The dates of fishery area-based closures around Johnston Atoll, Kingman Reef and Palmyra Atoll during the study period are summarized in Table 3. Johnston Atoll, a U.S. territory, is located in the central Pacific Ocean south of Hawaii (Fig 5). Palmyra Atoll and Kingman Reef, also U.S. territories, are the northernmost features of the Line Islands. Kingman and Palmyra are 61 km apart (Fig 5). A 1941 Executive Order established Naval Defensive Sea Areas within 0–3 nm around these areas in which commercial fishing was prohibited (the Naval Defensive Sea Area around Palmyra Atoll was eliminated in 1947) (Table 3) [120–121]. The Palmyra Atoll and Kingman Reef National Wildlife Refuges, established in 2001, ban commercial fishing within 12 nm from the mean low water line [122–124]. Established in 2009, the Pacific Remote Islands Marine National

Table 3. Dates of area-based fishery closures within the US EEZ adjacent to Johnston Atoll, Kingman Reef and Palmyra Atoll during the study period.

Start	End	Fishery Closures	Citations
4 March 1994[1]	17 January 2001	Johnston: 0–3 nm	[120–121]
		Kingman: 0–3 nm	
18 January 2001[2]	5 January 2009[3]	Johnston: 0–3 nm	[122–123]
		Kingman: 0–12 nm	
		Palmyra: 0–12 nm	
6 January 2009[3]	29 September 2014[4]	Johnston: 0–50 nm	[128, 130]
		Kingman: 0–50 nm	
		Palmyra: 0–50 nm	
30 September 2014[4]	31 October 2018[5]	Johnston: 0–200 nm	[129, 131]
		Kingman: 0–50 nm	
		Palmyra: 0–50 nm	

[1] Study period begins on 4 March 1994. A Naval Defensive Sea Area was established at Johnston Atoll in 1941 through Executive Order No. 8682 and remains in effect [120]. Naval Defensive Sea Areas were established at Kingman Reef and Palmyra Atoll in 1941. The Palmyra Naval Defensive Sea Area was eliminated in 1947. In 2000 the Navy transferred "custody and accountability for Kingman Reef" to the Department of the Interior [121, 124].

[2] Kingman Reef and Palmyra Atoll National Wildlife Refuges were established on 18 January 2001 [117–118].

[3] Presidential Proclamation 8336 establishing the Pacific Remote Islands Marine National Monument was signed on 6 January 2009 [128]. NMFS regulations implementing the ban on commercial fishing came into effect on 3 July 2013 [130].

[4] Presidential Proclamation 9173, signed on 29 September 2014, expanded the seaward margin of the Marine National Monument at Johnston Atoll out to 200 nm [129]. NMFS regulations implementing the ban on commercial fishing in the extended Monument boundary came into effect on 24 April 2015 [131].

[5] Study period ends 31 October 2018

https://doi.org/10.1371/journal.pone.0235129.t003

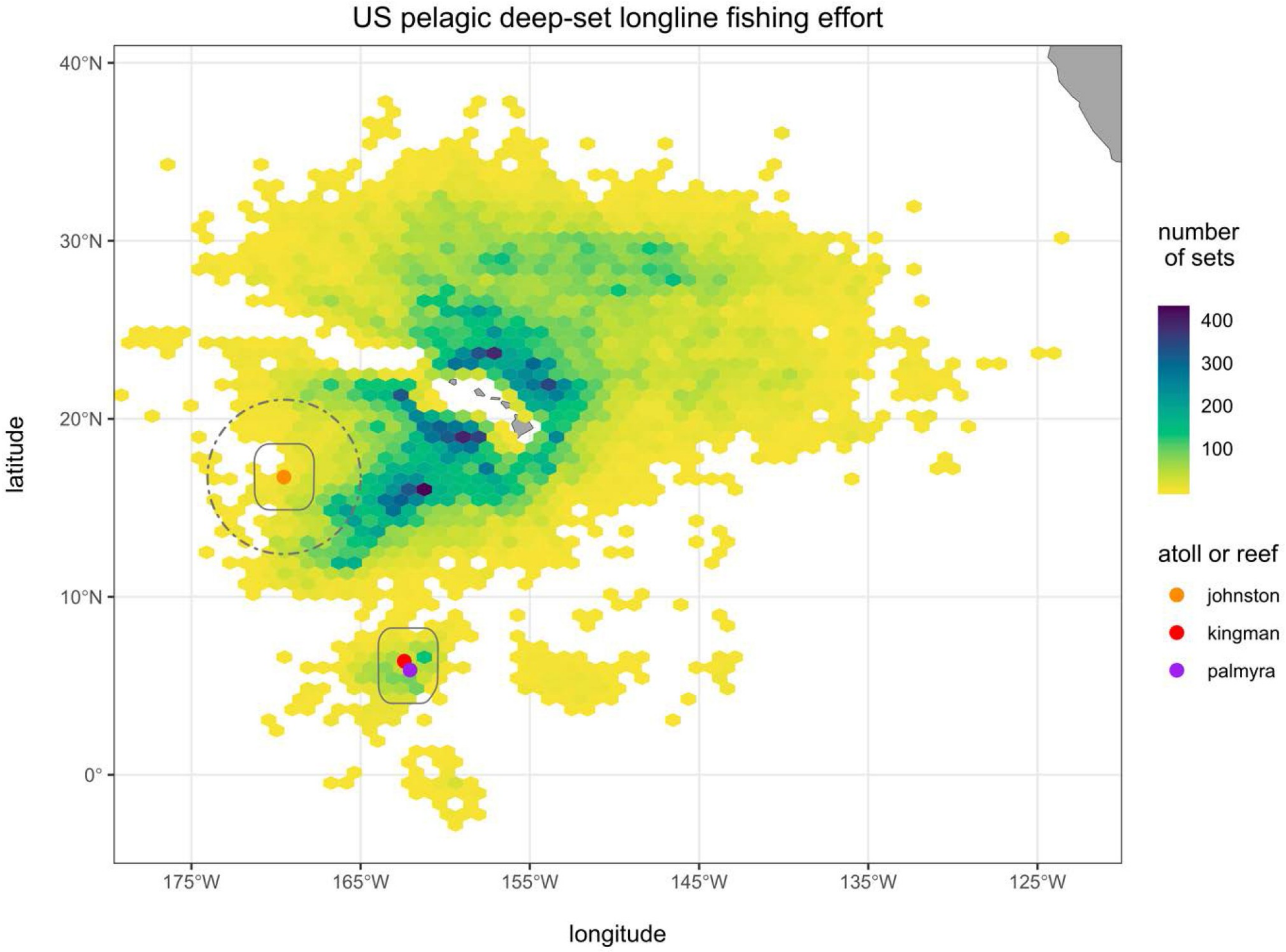

Fig 5. Spatial density trend in Hawaii tuna pelagic longline fishing effort (1994–2018) shown using hexagon binning with ≥ 50 sets per bin. The 110 nm seaward margin (treatment zone boundary) around Kingman/Palmyra and Johnston shown by a solid-line polygon. The 260 nm seaward margin (treatment zone boundary) around Johnston shown by a dashed-line circle.

https://doi.org/10.1371/journal.pone.0235129.g005

Monument includes these two Refuges within its boundaries, as well as the Johnston Atoll National Wildlife Refuge, established in 1926 [125–126]. The 2009 designation banned commercial fishing within 50 nm [127–128]. In 2014, the Monument was expanded out to the 200 nm seaward limit of the U.S. EEZ adjacent to Johnston [129].

Between 6 January 2009 and 3 July 2013, the period between the date of the proclamation that established the Pacific Remote Islands Marine National Monument and of the date that fisheries regulations implementing the proclamation came into effect, a very small number of sets (we cannot state the exact number due to US government data confidentiality requirements) occurred within the closed area around Kingman and Palmyra, and no fishing occurred in the closed area around Johnston. No Hawaii longline fishing occurred in the U.S. EEZ adjacent to Johnston between 30 September 2014 and 24 April 2015, the period between the date of the proclamation that expanded the Monument boundary around Johnston and the date that fisheries implementing regulations came into effect. As there was extremely little fishing effort within closed areas between the dates of designation and adoption of regulations prohibiting fishing, we used the designation dates, and not the dates of fishing regulations coming into effect, to define study period components.

For counterfactual modeling assessments, treatment zones were defined as the MPA fishery closure areas plus ‘spillover’ areas of 60 nm from the seaward margin of the MPA. We explored but could not employ smaller 30 nm spillover areas within counterfactual treatment zones due to inadequate sample sizes of fishing effort. A treatment zone of 0–110 nm from the mean low water line was employed for Kingman Reef/Palmyra Atoll and Johnston Atoll for the 50 nm MPAs (referred to as KP110 and J110, respectively). A 0–260 nm treatment zone was employed for Johnston Atoll for the 200 nm MPA (referred to as J260). Three control zones, the portion of the study area that was outside of treatment zones, were defined south, northwest and northeast of the Hawaiian archipelago employing a boundary roughly through the Hawaiian archipelago (running through 28.4167˚N, 178.3333˚W (Kure Atoll) and 19.5429˚N, 155.6659˚W (Hawaii Island), and a boundary along 150˚W, which is the boundary between the convention areas of the two Pacific Ocean tuna regional fisheries management organizations in the northern hemisphere [132–133].

Fig 5 shows the spatial distribution of effort during the study period, aggregated into ca. 1x1-degree hexagonal bins (hexbins), and shows the seaward margins of the three counterfactual treatment zones. Data records were removed that did not meet government confidentiality requirements for effort by three or more individual vessels within an aggregate. There were 494 sets made within 50 nm of Kingman and Palmyra and 1,132 sets made within 200 nm of Johnston, prior to MPA establishment. There were 62,985 sets made outside of these MPA zones. There were 1,192 sets within KP110, and 2,584 sets within J260 (of which 313 were between 0–110 and 2,271 were between 110–260 nm of Johnston). There were 8,889 sets made in the northeast, 27,145 in the northwest and 24,801 in the southern control zones (Fig 5). Not shown in order to meet confidentiality requirements, the spatial distribution of effort was dynamic, moving northeastward and away from the Johnston and Kingman/Palmyra areas over the study period. Prior to and after establishment of the Kingman/Palmyra 50 nm MPA, the Hawaii tuna longline fleet made 68 and 19 observed sets per year, respectively, within KP110. Similarly, prior to and after establishment of the 50 nm MPA at Johnston, the fleet made 18 and 4 observed sets per year, respectively, within J110. And, prior to and after establishment of the 200 nm MPA at Johnston, the fleet made 114 and 58 sets per year, respectively, within J260. Hence, a substantial decline in observed annual effort by the Hawaii tuna longline fishery occurred near the MPAs, within counterfactual treatment zones, after their establishment.

Statistical modeling approaches for counterfactual predictions

An important issue in conservation management is how to infer the causal ecological impact attributable to a specific policy intervention [134], such as the implementation of large pelagic MPAs [89]. We used the two-stage modelling approach advocated in Gilman et al. [20] that comprises (1) estimation of the zone-specific mean response for commonly used population-level metrics [135] such as species-specific catch rate, mean length, mean trophic level and so on using either generalized linear mixed model or GAMM structured regression models [136] with the appropriate response-specific likelihood and then (2) fitting a Bayesian structural time series model [137] to the predicted species-specific annual trends to derive the counterfactual prediction for each metric within each of the treatment (MPA) and reference (or control) zones. So, stage 1 focused on standardization of the fishery-dependent sourced catch and effort data [138] that was needed for causal inference modelling in the next stage. Stage 2 then focused on deriving the zone-specific counterfactual predictions using the model-standardized data to evaluate whether there was any temporal causal impact on the pelagic longline fishery that could be attributable to the Johnston and Palmyra/Kingman MPA expansions.

Supporting Information S1 Section in S1 File describes data sources for the observer program for Hawaii's deep-set pelagic longline fishery, sea surface temperature, monthly Pacific Decadal Oscillation index, Food and Agriculture Organization of the United Nations (FAO) Fish Price Index, bathymetric depth and TLc. S2 Section in S1 File presents methods for data standardization models for catch rates, mean length, mean TLc and Shannon-Wiener Index of diversity *H'* (also referred to as the Shannon Index), for calculating set-specific mean TLc, for the selection of species included in catch rate and length assessments, and for exploring the importance of effort standardization to account for any zone-specific inadequate sample sizes in estimating a species diversity response.

Evaluating social, conservation or management policy interventions using observational data is challenging and can lead to ambiguous conclusions [139]—especially if the intervention is nonrandomized, there are few treatment units affected by the intervention and there are multiple time-dependent outcome measures [140]. In our case, the MPA expansions (interventions) were prescribed (not random) and also binary (all or nothing), there were few MPAs to assess, and the data series were time-dependent. We confronted these challenges using a counterfactual prediction or potential outcomes framework [141–142] to infer if there was any temporal causal impact on the pelagic longline fishery attributable to MPA expansions in 2009. The counterfactual prediction is then the "unknown" or "unobserved" or inferred outcome of the response metric in the absence of an intervention, which in our case for instance is "what might have happened to the trend in annual bigeye tuna catch rates if MPA expansions had not occurred"? Counterfactual prediction-based approaches are increasingly used to infer temporal causal impacts in a wide range of policy evaluation contexts including public health [143], social policy [144], cigarette smoking bans [145], water conservation initiatives [146] and the impact on seafood markets of radioactive spills [147] or coastal hypoxia [148].

Using the regression-model-standardized response metrics, we constructed time series of the trends in the predicted mean annual metric such as bigeye tuna catch rates for 6 comparison zones: 3 MPA (treatment) zones and 3 references (control) zones. Treatment zones were (1) within 110 nm around Palmyra/Kingma, (2) with 110 nm around Johnston and (3) within 260 nm of Johnston. The reference zones of increasing distance from the MPAs considered important for estimating MPA impacts [135] were (1) the southern zone, (2) the northwest zone, and (3) the northeast zone. We then fit a Bayesian state-space or structural times series model [137, 140] with weakly informative regularizing priors to the zone-specific data using the `CausalImpact` R package [137]. We focus only on the 2009 intervention since the 2014 MPA expansions are too recent to be evaluated using this approach. The fitted structural time series model synthetic control series is then predicted well beyond the intervention (post-2009) to derive the counterfactual prediction (the temporal trend without any MPA). Here the synthetic control is a multivariate ensemble or composite comprising the 3 reference zone time series and potentially informative annual predictors that are not affected by the intervention such as the annual FAO Fish Price index [149] as a proxy for fisher behavior and macro-scale ocean/climate environmental drivers (see S1.2 Section in S1 File). The same structural time series model is also fitted simultaneously to the standardized response metric times series exposed to the intervention, for instance, the model-standardized bigeye tuna annual catch rate trends.

Any difference between the 2 simulated time series (the modelled output based on the standardized data series with 2009 intervention and the synthetic control or counterfactual prediction) is the temporal measure of any MPA-attributable impact. The posterior predictive draws or samples are then summarized in a counterfactual prediction plot that supports better understanding of the temporal evolution of any post-intervention effect [137], for instance, "was it abrupt or gradual, or temporary or permanent, or was there a delay in the response?" [150].

The posterior predictive samples can also be summarized as mean point estimates with 95% credible intervals such as for the posterior mean predictive probability of a causal effect and the magnitude of any causal effect [137].

This Bayesian state-space time series modelling approach also comprises a model averaging component via spike-and-slab regression to choose a minimal or sparse set of the reference controls and other predictors used to create the multivariate composite or the synthetic control [137], which minimizes the dependence of the counterfactual prediction on the references and predictors in the model. The importance of each reference and predictor contribution to the composite synthetic control series can be shown in an inclusion probability plot. Unlike the commonly used difference-in-differences approach [20, 151], the synthetic control approach to counterfactual prediction makes no assumptions about the data generating mechanism and is especially applicable for sparse data settings (few treatment units), unlike the closely related covariate matching methods (see the review in [140]). When there are a large number of treatment units (hundreds or thousands) in a policy intervention analysis, then it can be informative to combine the synthetic control method with unit-specific covariate matching in a Bayesian counterfactual prediction modelling framework [146].

Counterfactual model assessments were conducted for the 50 nm MPA around Kingman/Palmyra, and for the 50 nm and 200 nm MPAs around Johnston. Counterfactual model assessments were not conducted for the 3 nm closures at Johnston and Kingman because these two interventions did not occur during the study period (preventing a counterfactual analysis of time series data before the intervention occurred), and because these were relatively small closures. Sample sizes for the 12 nm MPAs were too sparse to support counterfactual model assessments.

Counterfactual model assessments for standardized catch rate responses were conducted for KP110, J110 and J260. As with the standardized catch rate counterfactual assessment, the counterfactual assessments for the mean length and mean TLc responses were conducted for KP110. However, only a single counterfactual assessment was conducted for the combined Johnston treatment zones, as the sample size for J110 was too sparse to support a separate assessment. S2 Section in S1 File describes the methods employed for the selection of species included in the counterfactual model assessments for the standardized catch rate (albacore *T. alalonga*, bigeye, and yellowfin tunas, blue shark, longnose lancetfish, striped marlin) study component. Mean length assessments were conducted for bigeye and yellowfin tunas. A single counterfactual modeling assessment was conducted to infer what the Shannon index within combined treatment zones would have been if the 2009 and 2014 MPA interventions had not occurred.

Comparison of responses within and outside of MPAs/treatment zones

We compared the abundance-adjusted Shannon diversity index and the catch rate of species of conservation concern between the MPAs (50 nm of Kingman/Palmyra, 200 nm of Johnston) and the rest of fishing grounds. We also compared annual mean TLc for sets occurring either inside or outside of redefined treatment zones (110 nm around Kingma/Palmyra, 260 nm around Johnston).

The methods described in S2 Section in S1 File for data standardization models for the Shannon diversity index and mean TLc were also employed for this component for these two attributes. Catch rates for five bycatch species with adequate sample sizes were fit to GAMMs with negative binomial in order to compare catch rates, an indicator of relative abundance, within future MPAs or outside of the MPAs. Here the predictors were zone (within and outside of MPAs), effort (log hooks per set), year, season (quarter), bait type, hook type and

hooks-per-float and with the 202 individual vessels as the intercepts-only random effect. These five species were: striped marlin and blue, bigeye thresher, oceanic whitetip and silky sharks. For those bycatch species of conservation concern with only limited sample sizes, we summed the species-specific catch and effort data for all years. Then we estimated the mean posterior catch rate and highest posterior density interval for each species using the `binom` R package [152] by sampling from a binomial likelihood with a Bayes-Laplace prior [153]. Those 4 species/groups were: albatross species (black-footed *Phoebastria nigripes*, Laysan *P. immutabilis*), olive Ridley sea turtle, shortfin mako shark and all odontocete species.

Supporting information

S1 File.
(PDF)

Acknowledgments

We thank journal editor Dr. Heather Patterson and two anonymous reviewers for their insightful and constructive comments. We acknowledge Eric Forney of the US National Marine Fisheries Service for vital assistance with a query of the Hawaii pelagic longline observer program database. We are grateful for assistance provided by Jarad Makaiau and Kevin Brindock of US National Marine Fisheries Service with compiling information on the dates of Hawaii longline area closures resulting from catch limits for bigeye tuna and false killer whales being reached. We thank Adrienne Egger of FAO and the Norwegian Seafood Council for providing the annual FAO fish price databases. The findings, conclusions and views expressed in this article are those of the authors and do not necessarily reflect the views or policies of their affiliated organizations, including the Western Pacific Regional Fishery Management Council and U.S. Fish and Wildlife Service.

Author Contributions

Conceptualization: Eric Gilman, Milani Chaloupka, Danielle L. Cantrell, Matt Merrifield.

Formal analysis: Eric Gilman, Milani Chaloupka, Mark Fitchett, Danielle L. Cantrell.

Investigation: Eric Gilman, Milani Chaloupka, Danielle L. Cantrell, Matt Merrifield.

Methodology: Eric Gilman, Milani Chaloupka, Mark Fitchett, Danielle L. Cantrell, Matt Merrifield.

Project administration: Eric Gilman, Milani Chaloupka.

Validation: Milani Chaloupka.

Visualization: Milani Chaloupka.

Writing – original draft: Eric Gilman, Milani Chaloupka, Mark Fitchett, Danielle L. Cantrell, Matt Merrifield.

Writing – review & editing: Eric Gilman, Milani Chaloupka, Mark Fitchett, Danielle L. Cantrell, Matt Merrifield.

References

1. Halpern B. The impact of marine reserves: Do reserves work and does reserve size matter? Ecol Appl. 2003; 13: S117–S137.

2. Roberts CM, Bohnsack JA, Gell F, Hawkins J, Goodridge R. Effects of marine reserves on adjacent fisheries. Science. 2001; 294:1920–1923. https://doi.org/10.1126/science.294.5548.1920 PMID: 11729316
3. Roberts C, Hawkins J, Gell F. The role of marine reserves in achieving sustainable fisheries. Phil Trans R Soc B. 2005; 360: 123−132. https://doi.org/10.1098/rstb.2004.1578 PMID: 15713592
4. Lester S, Halpern B, Grourud-Covert K., Lubchenco J., Ruttenberg B, Gaines S, et al. Biological effects within no-take marine reserves: a global synthesis. Mar Ecol Prog Ser. 2009; 384: 33−46.
5. Stewart G, Kaiser M, Côté I, Halpern B, Lester S, Bayliss H, et al. Temperate marine reserves: global ecological effects and guidelines for future networks. Conserv Lett. 2009; 2: 243–253.
6. Kaiser MJ, Hormbrey S, Booth J, Hinz H, Hiddink J. Recovery linked to life-history of sessile epifauna following exclusion of towed-mobile fishing gear. J Appl Ecol. 2018; 55: 1060–1070.
7. Micheli F, Amarasekare A, Bascompte J, Gerber L. Including species interactions in the design and evaluation of marine reserves: some insights from a predator–prey model. B Mar Sci. 2004; 74: 653–669.
8. Micheli F, Halpern BS, Botsford LW, Warner RR. Trajectories and correlates of community change in no-take marine reserves. Ecol Appl. 2004; 14: 1709-1723.
9. Le Quesne W, Codling E. Managing mobile species with MPAs: the effects of mobility, larval dispersal, and fishing mortality on closure size. ICES J Mar Sci. 2009; 66: 122–131.
10. Claudet J, Osenberg C, Domenici P, Badalamenti F, Milazzo M, Falcon J, et al. Marine reserves: fish life history and ecological traits matter. Ecol Appl. 2010; 20:830-839. https://doi.org/10.1890/08-2131.1 PMID: 20437967
11. Grüss A, Kaplan D, Hart D. Relative impacts of adult movement, larval dispersal and harvester movement on the effectiveness of reserve networks. PloS One. 2011; 6: e19960. https://doi.org/10.1371/journal.pone.0019960 PMID: 21611148
12. Edgar G., Stuart-Smith R, Willis T, Kininmonth S, Baker S, Banks S, et al. Global conservation outcomes depend on marine protected areas with five key features. Nature. 2014; 506: 216–220. https://doi.org/10.1038/nature13022 PMID: 24499817
13. Gill D., Mascia M, Ahmadia G, Glew L, Lester S, Barnes M, et al. Capacity shortfalls hinder the performance of marine protected areas globally. Nature. 2017: 543: 665–669. https://doi.org/10.1038/nature21708 PMID: 28329771
14. Gillespie K, Vincent A. Tropical invertebrate response to marine reserves varies with protection duration, habitat type, and exploitation history. Aquat Conserv. 2019; 29: https://doi.org/10.1002/aqc.3019.
15. Gannon P, Seyoum-Edjigu E, Cooper D, Sandwith T, Dias B, Palmer C, et al. Status and prospects for achieving Aichi Biodiversity Target 11: Implications of national commitments and priority actions. Parks. 2017; 23: 13–26.
16. CBD. Aichi Target 11. Decision X/2. Montreal: Convention on Biological Diversity; 2011.
17. UNGA. Transforming Our World: The 2030 Agenda for Sustainable Development. Resolution adopted by the General Assembly on 25 September 2015. A/RES/70/1. New York: United Nations General Assembly; 2015.
18. Kaiser MJ. Are marine protected areas a red herring or fisheries panacea? Can J Fish Aquat Sci. 2005; 62: 1194–1199.
19. Kaplan D, Chassot E, Grüss A, Fonteneau A. Pelagic MPAs: The devil is in the details. Trends Ecol Evol. 2010; 25: 62–63. https://doi.org/10.1016/j.tree.2009.09.003 PMID: 19818526
20. Gilman E, Kaiser M, Chaloupka M. Do static and dynamic marine protected areas that restrict pelagic fishing achieve ecological objectives? Ecosphere. 2019; 10: e02968.
21. Reimer M, Haynie A. Mechanisms matter for evaluating the economic impacts of marine reserves. J Environ Econ Manag. 2018; 88: 427–446.
22. Fraser K, Adams V, Pressey R, Pandolfi J. Impact evaluation outcomes in marine protected areas: A case study of the Great Barrier Reef Marine Park. Biol Conserv. 2019; 238: https://doi.org/10.1016/j.biocon.2019.07.030.
23. Baum JK, Myers RA, Kehler DG, Worm B, Harley SJ, Doherty PA. Collapse and conservation of shark populations in the northwest Atlantic. Science. 2003; 299: 389–92. https://doi.org/10.1126/science.1079777 PMID: 12532016
24. Myers R, Worm B. Rapid worldwide depletion of predatory fish communities. Nature. 2003; 423: 280–283. https://doi.org/10.1038/nature01610 PMID: 12748640
25. Burgess G, Beerkircher L, Cailliet G, Carlson J, Cortes E, Goldman K, et al. Is the collapse of shark populations in the Northwest Atlantic Ocean and Gulf of Mexico real? Fisheries. 2005; 30:19–26

26. Hampton J, Sibert J, Kleiber P, Maunder M, Harley S. Decline of Pacific tuna populations exaggerated? Nature. 2005; 434: E1–E2. https://doi.org/10.1038/nature03581 PMID: 15858533
27. Ward P, Myers R. Shifts in open-ocean fish communities coinciding with the commencement of commercial fishing. Ecology. 2005; 86: 835–847.
28. Hall M, Alverson D, Metuzal K. By-catch: problems and solutions. Mar Pollut Bull. 2000; 41: 204–219.
29. Gilman E. Bycatch governance and best practice mitigation technology in global tuna fisheries. Mar Policy. 2011; 35: 590–609.
30. Branch T, Lobo A, Purcell S. Opportunistic exploitation: an overlooked pathway to extinction. Trends Ecol Evolut. 2013; 28: 409–413.
31. Heino M, Pauli B, Dieckmann U. Fisheries-induced evolution. Annu Rev Ecol Evol Syst. 2015; 46: 461–480.
32. Hollins J, Thambithurai D, Köeck B, Crespel A, Bailey D, Cooke S, et al. A physiological perspective on fisheries-induced evolution. Evol Appl. 2018; 11: 561–576. https://doi.org/10.1111/eva.12597 PMID: 29875803
33. Dagorn L, Holland K, Restrepo V, Moreno G. Is it good or bad to fish with FADs? What are the real impacts of the use of drifting FADs on pelagic marine ecosystems? Fish Fish. 2013; 14: 391–415.
34. Sempo G, Dagorn L, Robert M, Deneubourg J. Impact of increasing deployment of artificial floating objects on the spatial distribution of social fish species. J Appl Ecol. 2013; 50: 1081–1092.
35. Gilman E, Bigler B, Muller B, Moreno G, Largacha E, Hall M, et al. Methods to Identify Ownership and Track the Position of Drifting Fish Aggregating Devices Used by Tuna Purse Seine Fisheries with Reference to FAO's Draft Guidelines on the Marking of Fishing Gear. FAO Fisheries Circular 1163. Rome: Food and Agriculture Organization of the United Nations; 2018.
36. Pace M, Cole J, Carpenter S, Kitchell J. Trophic cascades revealed in diverse ecosystems. Trends Ecol Evol. 1999; 14: 483–488. https://doi.org/10.1016/s0169-5347(99)01723-1 PMID: 10542455
37. Baum J, Worm B. Cascading top-down effects of changing oceanic predator abundances. J Anim Ecol. 2009; 78: 699–714. https://doi.org/10.1111/j.1365-2656.2009.01531.x PMID: 19298616
38. Polovina J, Abecassis M, Howell E, Woodworth P. Increases in the relative abundance of mid-trophic level fishes concurrent with declines in apex predators in the subtropical North Pacific, 1996-2006. Fishery Bulletin. 2009; 107: 523–531.
39. Daskalov G, Grishin A, Rodionov S, Mihneva V. Trophic cascades triggered by overfishing reveal possible mechanisms of ecosystem regime shifts. P Natl Acad Sci USA. 2007; 104: 10518–10523.
40. Mollmann C, Diekmann R, Muller-Karulis B, Kornilovs G, Plikshs M, Axe P. Reorganization of a large marine ecosystem due to atmospheric and anthropogenic pressure: a discontinuous regime shift in the Central Baltic Sea. Glob Change Biol. 2009; 15: 1377–1393.
41. Crespo G, Dunn D. A review of the impacts of fisheries on open-ocean ecosystems. ICES J Mar Sci. 2017; 74: 2283–2297.
42. Pereira H, Leadley P, Proença V, Alkemade R, Scharlemann J, Fernandez-Manjarrés J, et al. Scenarios for global biodiversity in the 21st century. Science. 2010; 330: 1496–1501. https://doi.org/10.1126/science.1196624 PMID: 20978282
43. Halpern B, Frazier M, Potapenko J, Casey K, Koenig K, Longo C, et al. Spatial and temporal changes in cumulative human impacts on the world's ocean. Nat Commun. 2015; 6: https://doi.org/1038/ncomms8615
44. Botsford L, Micheli F, Hastings A. Principles for the design of marine reserves. Ecol Appl. 2003; 13: S25–S31.
45. Worm B, Lotze HK, Myers RA. Predator diversity hotspots in the blue ocean. PNAS. 2003; 100: 9884–9888. https://doi.org/10.1073/pnas.1333941100 PMID: 12907699
46. Morato T, Varkey DA, Damaso C, Machete M, Santos M, Prieto R, et al. Evidence of a seamount effect on aggregating visitors. Mar Ecol Prog Ser. 2008; 357: 23–32.
47. Morato T, Hoyle S, Allain V, Nicol S. Seamounts are hotspots of pelagic biodiversity in the open ocean. PNAS Early Edition. 2010; www.pnas.org/cgi/doi/10.1073/pnas.0910290107.
48. Gilman E, Chaloupka M, Read A, Dalzell P, Holetschek J, Curtice C. Hawaii longline tuna fishery temporal trends in standardized catch rates and length distributions and effects on pelagic and seamount ecosystems. Aquat Conserv. 2012; 22: 446–488.
49. Ward P. Empirical estimates of historical variations in the catchability and fishing power of pelagic longline fishing gear. Rev Fish Biol Fisheries. 2008; 18: 409–426.
50. Kuriyama P, Branch T, Hicks A, Harms J, Hamel O. Investigating three sources of bias in hook-and-line surveys: survey design, gear saturation, and multispecies interactions. Can J Fish Aquat Sci. 2019; 76: 192–207.

51. McKechnie S, Pilling G, Hampton J. Stock Assessment of Bigeye Tuna in the Western and Central Pacific Ocean. Noumea, New Caledonia: The Pacific Community; 2017

52. National Institute of Fisheries Science. National Report of the Republic of Korea. ISC/19/Plenary/07. Shimizu, Japan: International Scientific Committee for Tuna and Tuna-like Species in the North Pacific Ocean; 2019.

53. Rijnsdorp A, Piet G, Poos J. Effort allocation of the Dutch beam trawl fleet in response to a temporarily closed area in the North Sea. In: ICES. International Council for the Exploration of the Sea Meeting 2001/N:01. Copenhagen: International Council for the Exploration of the Sea; 2001. pp. 17–25.

54. Abbott J, Haynie A. What are we protecting? Fisher behavior and the unintended consequences of spatial closures as a fishery management tool. Ecol Appl. 2012; 22: 762–777. https://doi.org/10.1890/11-1319.1 PMID: 22645809

55. Gilman E, Chaloupka M, Dagorn L, Hall M, Hobday A, Musyl M, et al. Robbing Peter to pay Paul: Replacing unintended cross-taxa conflicts with intentional tradeoffs by moving from piecemeal to integrated fisheries bycatch management. Rev Fish Biol Fisher. 2019b; 29: 93–123.

56. Murray K, Read A, Solow A. The use of time/area closures to reduce bycatches of harbour porpoises: lessons from the Gulf of Maine sink gillnet fishery. J Cetac Res Manage. 2000; 2: 135–141.

57. Suuronen P, Jounela P, Tschernij V. Fishermen responses on marine protected areas in the Baltic cod fishery. Mar Policy. 2010; 34: 237–243.

58. Sibert J, Senina I, Lehodey P, Hampton J. Shifting from marine reserves to maritime zoning for conservation of Pacific bigeye tuna (Thunnus obesus). PNAS. 2012; 109: 18221–18225. https://doi.org/10.1073/pnas.1209468109 PMID: 23064639

59. Boyce J. An economic analysis of the fisheries bycatch problem. J Environ Econ Manag. 1996; 31: 314–336.

60. Litvinov F. Aggregations of large pelagic sharks above seamounts. In: Pitcher T, Morato T, Hart P, Clark M, Haggan N, Santos R, editors. Seamounts: Ecology, Fisheries and Conservation. Oxford: Blackwell Science; 2007. pp. 202–206.

61. Amorim P, Figueiredo M, Machete M, Morato T, Martins A, Santos R. Spatial variability of seabird distribution associated with environmental factors: A case study of marine Important Bird Areas in the Azores. ICES J Mar Sci. 2009; 66: 29–40.

62. Parrish F. Do monk seals exert top-down pressure in subphotic ecosystems? Mar Mammal Sci. 2009; 25: 91–106.

63. Fonteneau A. Sea mounts and tuna in the tropical eastern Atlantic. Aquat Living Resour. 1991; 4: 13–25.

64. Itano D, Holland K. Movement and vulnerability of bigeye (Thunnus obesus) and yellowfin (Thunnus albacares) in relation to FADs and natural aggregation points. Aquat Living Resour. 2000; 13: 213–223.

65. Sibert J, Holland K, Itano D. Exchange rates of yellowfin and bigeye tunas and fishery interaction between Cross seamount and nears-shore FADs in Hawaii. Aquat Living Resour. 2000; 13: 225–232.

66. Adam M, Sibert J, Itano D, Holland K. Dynamics of bigeye (Thunnus obesus) and yellowfin (T. albacares) tuna in Hawaii's pelagic fisheries: analysis of tagging data with a bulk transfer model incorporating size-specific attrition. Fish Bull. 2003; 101: 215–228.

67. Richardson A, Downes K, Nolan E, Brickle P, Brown J, Weber N, et al. Residency and reproductive status of yellowfin tuna in a proposed large-scale pelagic marine protected area. Aquat Conserv. 2018; 28: 1308–1316.

68. Morato T, Watson R, Pitcher TJ, Pauly D. Fishing down the deep. Fish Fish. 2006; 7: 24−34.

69. Swartz W, Sala E, Tracey S, Watson R, Pauly D. The spatial expansion and ecological footprint of fisheries (1950 to present). PLoS ONE. 2010; 5: e15143. https://doi.org/10.1371/journal.pone.0015143 PMID: 21151994

70. Pauly D, Christensen V, Dalsgaard J, Froese R, Torres F. Fishing down marine food webs. Science. 1998; 279: 860−863. https://doi.org/10.1126/science.279.5352.860 PMID: 9452385

71. Essington T, Beaudreau AH, Wiedenmann J. Fishing through marine food webs. Proc Natl Acad Sci USA. 2006; 103: 3171−3175. https://doi.org/10.1073/pnas.0510964103 PMID: 16481614

72. Hall M. On bycatches. Rev Fish Biol Fisher. 1996; 6: 319–352.

73. Garcia S., Kolding J., Rice J., et al. (2012) Reconsidering the consequences of selective fisheries. Science 335: 1045–1047. https://doi.org/10.1126/science.1214594 PMID: 22383833

74. Mackey R, Currie D. The diversity–disturbance relationship: is it generally strong and peaked? Ecology. 2001; 82: 3479–3492.

75. Hughes A, Byrnes J, Kimbro D, Stachowicz J. Reciprocal relationships and potential feedbacks between biodiversity and disturbance. Ecol Lett. 2007; 10: 849–864. https://doi.org/10.1111/j.1461-0248.2007.01075.x PMID: 17663718

76. Hughes R. Disturbance and diversity: An ecological chicken and egg problem. Nature Education Knowledge. 2010; 3: 48.

77. Garofalo G, Fiorentino F, Gristina M, Cusumano S, Sinacori G. Stability of spatial pattern of fish species diversity in the Strait of Sicily (central Mediterranean). Hydrobiologia. 2007; 580: 117–124.

78. Blyth-Skyrme R, Kaiser M, Hiddink J, Edwards-Jones G, Hart P. Conservation benefits of temperate marine protected areas: variation among fish species. Conservation Biology. 2006; 20: 811–820. https://doi.org/10.1111/j.1523-1739.2006.00345.x PMID: 16909574

79. Dueri S, Maury O. Modelling the effect of marine protected areas on the population of skipjack tuna in the Indian Ocean. Aquat Living Resour. 2013; 26: 171–178.

80. SCRFA. Science and Conservation of Fish Aggregations. Search Database. Hong Kong: The University of Hong Kong; 2019.

81. Collette B, Carpenter K, Polidoro B, Juan-Jorda M, Boustany A, Die D, et al. High value and long life–Double jeopardy for tunas and billfishes. Science 2011; 333: 291–292. https://doi.org/10.1126/science.1208730 PMID: 21737699

82. Hernández C, Witting J, Willis C, Thorrold S, Llopiz J, Rotjan R. Evidence and patterns of tuna spawning inside a large no-take Marine Protected Area. Sci Rep 2019; 9: 10772. https://doi.org/10.1038/s41598-019-47161-0 PMID: 31341251

83. Myers R, Bowen K, Barrowman N. Maximum reproductive rate of fish at low population sizes. Can J Fish Aquat Sci 1999; 56: 2404–2419.

84. Essington T. Trophic cascades in open ocean ecosystems. In: Terborgh J, Estes J, editors. Trophic cascades. predators, prey and the changing dynamics of nature. Washington, D.C.: Island Press; 2010. p. 91–106.

85. Moffitt E, Botsford L, Kaplan D, O'Farrell M. Marine reserve networks for species that move within a home range. Ecol Appl. 2009; 19:1835–1847. https://doi.org/10.1890/08-1101.1 PMID: 19831073

86. Graham R, Witt M, Castellanos D, Remolina F, Maxwell S, Godley B, et al. Satellite tracking of manta rays highlights challenges to their conservation. PLoS ONE. 2012; 7: e36834. https://doi.org/10.1371/journal.pone.0036834 PMID: 22590622

87. Sibert J, Hampton J. Mobility of tropical tunas and the implications for fisheries management. Mar Policy. 2003; 27: 87–95.

88. Gunn J., Hampton J, Evans K, Clear N, Patterson T, Bigelow K, et al. Migration and habitat preferences of bigeye tuna, Thunnus obesus, on the east coast of Australia–a project using archival and conventional tags to determine key uncertainties in the species stock structure, movement dynamics and CPUE trends. ISBN 1 876996 94 3. Canberra, Australia: CSIRO Marine Research; 2005.

89. Kenchington R, Kaiser M, Boerder K. MPAs, fishery closures and stock rebuilding. In: Garcia S, Ye Y, editors. Rebuilding of Marine Fisheries Part 2: Case Studies. Fisheries and Aquaculture Technical Paper No. 630/2. Rome: Food and Agriculture Organization of the United Nations; 2018. pp. 182–216.

90. Game E, Grantham H, Hobday A, Pressey R, Lombard A, Beckley L, et al. Pelagic protected areas: the missing dimension in ocean conservation. Trends Ecol Evol. 2009; 24: 360–369. https://doi.org/10.1016/j.tree.2009.01.011 PMID: 19324450

91. Halpern B, Lester S, Kellner J. Spillover from marine reserves and the replenishment of fished stocks. Environ Conserv. 2009; 36: 268–276.

92. Boerder K, Bryndum-Buchholz A, Worm B. Interactions of tuna fisheries with the Galapagos Marine Reserve. Mar Ecol Prog Ser. 2017; 585: 1–15.

93. Bucaram S, Hearn A, Trujillo A, Rentería W, Bustamante R, Morán G, et al. Assessing fishing effects inside and outside an MPA: The impact of the Galapagos Marine Reserve on the Industrial pelagic tuna fisheries during the first decade of operation. Mar Policy. 2018; 87: 212–225.

94. White T, Carlisle A, Kroodsma D, Block B, Casagrandi R, De Leo G, et al. Assessing the effectiveness of a large marine protected area for reef shark conservation. Bio Cons. 2017; 207: 64–71.

95. Chan H, Pan M. Spillover effects of environmental regulation for sea turtle protection in the Hawaii longline swordfish fishery. Mar Res Econ. 2016; 31: 259–279.

96. Carreon B. Palau's marine sanctuary backfires, leading to increased consumption of reef fish. Pacific nation's protected zone has led to commercial tuna fishing vessels leaving the country. The Guardian. 2020; 26 February: https://www.theguardian.com/world/2020/feb/27/palaus-marine-sanctuary-backfires-leading-to-increased-consumption-of-reef-fish.

97. Diamond S, Kleisner K, Duursma D, Wang Y. Designing marine reserves to reduce bycatch of mobile species: a case study using juvenile red snapper (Lutjanus campechanus). Can J Fish Aquat Sci. 2010; 67: 1335–1349.

98. Hobday A, Hartog J, Timmiss T, Fielding J. Dynamic spatial zoning to manage southern bluefin tuna (Thunnus maccoyii) capture in a multi-species longline fishery. Fish Oceanogr. 2010; 19: 243–253.

99. Horwood JW, Nichols J, Milligan S. Evaluation of closed areas for fish stock conservation. J Appl Ecol. 1998; 35: 893–903.

100. Hilborn R, Stokes TK, Maguire J, Smith A, Botsford L, Mangel M, et al. When can marine reserves improve fisheries management? Ocean Coast. Manag. 2004; 47: 197–205.

101. Hilborn R. Marine biodiversity needs more than protection. Nature. 2016; 535: 224–226. https://doi.org/10.1038/535224a PMID: 27411619

102. Leenhardt P, Cazalet B, Salvat B, Claudet J, Feral F. The rise of large-scale marine protected areas: Conservation or geopolitics? Ocean Coast Manage. 2013; 85: 112–118.

103. Sala E, Lubchenco J, Grorud-Colvert K, Novelli C, Roberts C, Sumaila U. Assessing real progress towards effective ocean protection. Mar Policy. 2018; 91: 11–13.

104. Jantke K, Jones K, Allan J, Chauvenet A, Watson J, Possingham H. Poor ecological representation by an expensive reserve system: evaluating 35 years of marine protected area expansion. Conserv Lett. 2018; 11: e12584.

105. Visconti P, Butchart S, Brooks T, Langhammer P, Marnewick D, Vergara S, et al. Protected area targets post-2020. Science. 2019; 364: 239–241. https://doi.org/10.1126/science.aav6886 PMID: 30975769

106. Pennino M, Paradinas I, Illian J, Munoz F, Bellido J, Lopez-Quilez A, et al. Accounting for preferential sampling in species distribution models. Ecol Evol. 2018; 9: 653–663. https://doi.org/10.1002/ece3.4789 PMID: 30680145

107. Spear LB, Ainley DG, Walker W. Foraging dynamics of seabirds in the eastern tropical Pacific Ocean. Stud Avian Biol. 2007; 35: 1−99.

108. Cury P, Boyd I, Bonhommeau S, Anker-Nilssen T, Crawford R, Furness R, et al. Global seabird response to forage fish depletion—one-third for the birds. Science. 2011; 334: 1703–1706. https://doi.org/10.1126/science.1212928 PMID: 22194577

109. Sherley R, Winder H, Altwegg R, van der Lingen C, Votier S, Crawford R. Bottom-up effects of a no-take zone on endangered penguin demographics. Biol Lett. 2015; 11: 20150237. https://doi.org/10.1098/rsbl.2015.0237 PMID: 26156127

110. Sherley B, Barham B, Barham P, Campbell K, Crawford R, Grigg J, et al. Bayesian inference reveals positive but subtle effects of experimental fishing closures on marine predator demographics. Proc R Soc B. 2018; 285: 20172443. https://doi.org/10.1098/rspb.2017.2443 PMID: 29343602

111. Griffiths J, Kadin M, Nascimento F, Tamelander T, Tornroos A, Bonaglia S, et al. The importance of benthic-pelagic coupling for marine ecosystem functioning in a changing world. Glob Change Biol. 2017; 23: 2179–2196.

112. Roberts C, Branch G, Bustamante R, Castilla J, Dugan J, Halpern B, et al. Application of ecological criteria in selecting marine reserves and developing reserve networks. Ecol. Appl. 2003; 13: S215–S228.

113. Dunn D. (Ed.). Ecologically or Biologically Significant Areas in the Pelagic Realm: Examples and Guidelines–Workshop Report. ISBN 978-2-8317-1426-4. Gland, Switzerland: IUCN; 2011.

114. Gilman E, Dunn D, Read A, Warner R, Hyrenbach K. Designing criteria suites to identify sites and networks of high value across manifestations of biodiversity. Biodivers Conserv. 2011; 20: 3363–3383.

115. Fulton E, Smith A. Lessons learnt from a comparison of three ecosystem models for Port Philip Bay, Australia. Afr J Mar Sci. 2004; 26: 219–243.

116. Link J. Translating ecosystem indicators into decision criteria. ICES J Mar Sci. 2005; 62: 569–76.

117. Borenstein M, Hedges L, Higgins J, Rothstein H. Introduction to Meta-analysis. West Sussex, UK: Wiley Press; 2009.

118. Koricheva J, Gurevitch J, Mengersen K. Handbook of meta-analysis in ecology and evolution. Princeton: Princeton University Press; 2013

119. Mosqueira I, Côté I, Jennings S, Reynolds J. Conservation benefits of marine reserves for fish populations. Anim Conserv. 2000; 4: 321–332.

120. US Government. Executive Order 8682. Establishing naval defensive sea areas around and naval airspace reservations over the islands of Palmyra, Johnston, Midway, Wake, and Kingman Reef. Federal Register. 1941; 6: 1015.

121. US Government. Executive Order 9881. Discontinuing the Palmyra Island Naval Airspace Reservation and the Palmyra Island Naval Defensive Sea Area. Federal Register. 1947; 12: 5325.

122. USFWS. Establishment of the Kingman Reef National Wildlife Refuge. Federal Register. 2001; 66: 7660.

123. USFWS. Establishment of the Palmyra Atoll National Wildlife Refuge. Federal Register. 2001; 66: 7760–1.

124. USFWS. Palmyra Atoll National Wildlife Refuge and Kingman Reef National Wildlife Refuge Federal Register. 2007; 72: 25771–25773.

125. US Government. Executive Order 4467. Establishing Johnston Island Reservation. Washington, DC: The White House, U.S. Government; 1926.

126. US Government. Proclamation 2416. Changing the names of certain Federal Wildlife Refuges. Federal Register. 1940; 5: 2677.

127. Secretary of the Interior. Delegation of Management Responsibility for the Pacific Remote Islands Marine National Monument, Rose Atoll National Monument and the Marianas Trench Marine National Monument. Order No. 3284. Washington, D.C.: U.S. Department of the Interior; 2009.

128. US Government. Establishment of the Pacific Remote Islands Marine National Monument. Presidential Proclamation 8336. Washington, DC: The White House; 2009

129. U.S. Government. Presidential Proclamation 9173. Pacific Remote Islands Marine National Monument Expansion. Federal Register. 2014; 79: 58645–58653.

130. NMFS. Western Pacific fisheries; fishing in the Marianas Trench, Pacific Remote Islands, and Rose Atoll Marine National Monuments. Federal Register. 2013; 78: 32996–33007.

131. NMFS. Pacific island fisheries; Pacific Remote Islands Marine National Monument Expansion. National Marine Fisheries Service. Federal Register. 2015; 80: 15693–15695.

132. IATTC. Inter-American Tropical Tuna Commission Convention for the Strengthening of the Inter-American Tropical Tuna Commission Established by the 1949 Convention between the United States of America and the Republic of Costa Rica ("Antigua Convention"). La Jolla, USA: Inter-American Tropical Tuna Commission; 2003.

133. WCPFC. Convention on the Conservation and Management of Highly Migratory Fish Stocks in the Western and Central Pacific Ocean. Kolonia, Federated States of Micronesia: Western and Central Pacific Fisheries Commission; 2000.

134. Ferraro P, Hanauer M. Advances in measuring the environmental and social impacts of environmental programs. Annu Rev Env Resour. 2014; 39: 495–517.

135. Mascia M, Fox H, Glew L, Ahmadia G, Agrawal A, Barnes M, et al. A novel framework for analyzing conservation impacts: evaluation, theory, and marine protected areas. Ann NY Acad Sci. 2017: 1399: 93–115.

136. Wood S. Generalized Additive Models: an introduction with R. Boca Raton: Chapman and Hall/CRC; 2006.

137. Brodersen K, Gallusser F, Koehler J, Remy N, Scott S. Inferring causal impact using Bayesian structural time-series models. Ann Appl Stat. 2015; 9: 247–274.

138. Maunder M, Punt A. Standardizing catch and effort data: a review of recent approaches. Fish Res. 2004; 70: 141–159.

139. Pawson R, Wong G, Owen L. Known knowns, known unknowns, unknown unknowns: the predicament of evidence-based policy. Am J Eval. 2011; 32: 518–546.

140. Samartsidis P, Seaman S, Presanis A, Hickman M, de Angelis D. Assessing the causal effect of binary interventions from observational panel data with few treated units. Stat Sci. 2019; 34: 486–503.

141. Hofler M. Causal inference based on counterfactuals. BMC Med Res Methodol. 2005; 5: 28. https://doi.org/10.1186/1471-2288-5-28 PMID: 16159397

142. Rubin D. Causal inference using potential outcomes: design, modeling, decisions. Journal of the American Stat Assoc. 2005; 100: 322–331.

143. Bruhn C, Hetterich S, Shuck-Paim C, Kürüm E, Taylor R, Lustig R, et al. Estimating the population-level impact of vaccines using synthetic controls. P Natl Acad Sci USA. 2017; 114: 1524–152.

144. de Vocht F, Tilling K, Pliakas T, Angus C, Matt M, Brennan A, et al. The intervention effect of local alcohol licensing policies on hospital admission and crime: a natural experiment using a novel Bayesian synthetic time-series method. J Epidemiol Commun H. 2017; 71: 912–918.

145. Pinilla J, Negrín M, González López-Valcárcel B, Vázquez-Polo F. Using a Bayesian structural time-series model to infer the causal impact on cigarette sales of partial and total bans on public smoking. J Econ Stat. 2018; 238: 423–439.

146. Schmitt E, Tull C, Atwater P. Extending Bayesian structural time-series estimates of causal impact to many-household conservation initiatives. Ann Appl Stat. 2018; 12: 2517–2539.

147. Wakamatsu H, Miyata T. Do radioactive spills from the Fukushima disaster have any influence on the Japanese seafood market? Mar Res Econ. 2016; 31: 27–45.

148. Smith M, Zhang J, Coleman F. Effectiveness of marine reserves for large-scale fisheries management. Can J Fish Aquat Sci. 2006; 63: 153–164.

149. Tveteras S, Asche F, Bellemare M, Smith M, Guttormsen A, Lem A, et al. Fish Is Food—The FAO's Fish Price Index. PLoS ONE. 2012; 7: e36731. https://doi.org/10.1371/journal.pone.0036731 PMID: 22590598

150. Fujitani M, Fenichel E, Torre J, Gerber L. Implementation of a marine reserve has a rapid but short-lived effect on recreational angler use. Ecol Appl. 2012; 22: 597–605. https://doi.org/10.1890/11-0603.1 PMID: 22611857

151. Larsen A, Meng K, Kendall B. Causal analysis in control-impact ecological studies with observational data. Methods Ecol. 2019; 10: 924–934

152. Dorai-Raj S. binom: Binomial confidence intervals for several parameterizations. R package version 1.1–1. Comprehensive R Archive Network (CRAN). 2014; https://CRAN.R-project.org/package=binom.

153. Tuyl F, Gerlach R, Mengersen K. Comparison of Bayes-Laplace, Jeffreys, and other priors: the case of zero events. Am Stat. 2008; 62: 40–44.
